# On multicomponent stress–strength reliability for progressively censored logistic exponential model

**DOI:** 10.1016/j.aej.2025.06.004

**Published:** 2025-08

**Authors:** Amulya Kumar Mahto, Kumar Abhishek, Yogesh Mani Tripathi, Oluwafemi Samson Balogun, Yusra A. Tashkandy, Mahmoud E. Bakr

**Affiliations:** a Mehta Family School of Data Science and Artificial Intelligence, Indian Institute of Technology Guwahati, Assam 781039, India; b Department of Mathematics, Indian Institute of Technology Patna, Bihar 801106, India; c Department of Computing, Faculty of Science Forestry and Technology, University of Eastern Finland, Kuopio, Finland; d Department of Statistics and Operations Research, College of Science, King Saud University, P.O. Box 2455, Riyadh 11451, Saudi Arabia

**Keywords:** 62F10, 62F15, 62N02, Bayes estimation, Credible intervals, Lindley approximation, Maximum likelihood estimation, Progressive censoring scheme

## Abstract

The recent era of technological revolution led to the design and development of reliable products with multiple components for multiple functionality which produced a tough situation for manufacturers to test reliability of such products before launching their products to the real world market. This paper considers reliability estimation for a multicomponent stress–strength (MSS) model under progressively censored data. The classical and Bayesian estimation procedures are employed to evaluate point and interval estimators of the reliability when the failure pattern of the stress and strength components are modeled using the highly flexible logistic exponential distributions with a common shape parameter. When this common parameter is unknown, maximum likelihood estimators of MSS reliability are derived, and the corresponding asymptotic interval is also developed. Further Bayesian point estimators are obtained using two different techniques namely Lindley approximation and Markov chain Monte Carlo. In sequel credible intervals are also constructed. Similar inferences for the considered reliability are also derived having common shape parameter known. Further, an extensive simulation study is conducted and observations are noted. At the end, to illustrate the proposed methods, a real data set is also analyzed.

## 1 Introduction

Humongous technological advancements can be seen in every aspect of human life these days. From a smartwatch to large manufacturing equipment, from smart shoes to high-speed trains, from needles to high-precision medical equipment, every field of human life has seen very interesting changes to make life more comfortable. With all these advancements, the demand and desire for better than existing systems/products/units has increased exponentially. The same demand and desire have increased a more competitive market for manufacturers, which has made manufacturing more stressful but has also provided an opportunistic sense. A major effort is needed to understand the reliability of the systems/products/units developed with some extent of strength to be used under various possible stress conditions. Therefore, before bringing newly developed systems/products/units to the open market, careful reliability assessment is an important task for manufacturers.

In reliability analysis, the study of system reliability with some specific strength working under stress has been of great interest for a long time, with an obvious observation that a system ceases to function when the enforced stress becomes more than the actual strength of the system. This scenario is very frequent in almost all types of systems, such as mechanical, electrical, and medical equipment. Suppose random variables X and Y represent strength and stress components of a system under experimentation, then reliability of the system is R=P(X>Y). Inference with MSS reliability has been explored widely. Some of the early works include the pioneering work by Birnbaum [1]. Since then, a lot of developments has been made on MSS inference problems under different distributions using both classical and Bayesian techniques. Mukherjee and Sharan [2] derived inference for failure probability considering bivariate normal distributions. Further, Hanagal [3] explored the reliability estimation of the bivariate Pareto stress–strength model, and the list is long. Among some of the recent works we refer to Rao et al. [4], Qayoom et al. [5], Fulment et al. [6], Ahmad et al. [7], Wang et al. [8], Hussam and ALMetwally [9], Kohansal and Nadarajah [10], Hussam et al. [11], Rezaei et al. [12], Alosey and Gemeay [13], Saini et al. [14], Ragab and Elgarhy [15], BuHamra et al. [16], Agiwal [17], Alghamdi et al. [18], Kumari et al. [19], El-Alosey et al. [20], Asadi et al. [21], Tashkandy et al. [22], Sindhu et al. [23], Hassan and Morgan [24], Moheb et al. [25], Milošević and Stanojević [26], Shafiq et al. [27] and Lone et al. [28] and many more are there in the list.

With advancements in various technologies, the functionality of systems is highly increased, further making customers more desirous for such multi-functionality systems. Therefore, the systems are equipped with multiple multi-functional components for customer satisfaction. Such systems are termed as multi-component systems. The study of the reliability of such systems under stress–strength setup is a task of prime interest to manufacturers before they bring their products to market. Consider a system with k strength components being independent and are also identically distributed and a common stress component working on them, then the multi-component system functions when s(1≤s≤k) or a higher number of components operate simultaneously. The reliability study of such systems has been studied with great interest in recent years, but the model was first proposed by Bhattacharyya and Johnson [29] as

(1) Rs,k=P[at leastsof(X1,X2,…,Xk)exceedY]=∑p=skkp∫−∞∞[1−Fx(y)]p[Fx(y)]k−pdFy(y),

where $X_{1},X_{2},\ldots ,X_{k}$ denote identical strength components with distribution function $F_{X}\left(.\right)$ and are subjected to a common stress Y having distribution given by $F_{Y}\left(.\right)$. The recent works considering MSS components include [30], who considered the estimation of reliability when data are derived from Burr XII distributions, and Kizilaslan and Nadar [31] when data are derived from Weibull distribution. Nadar and Kızılaslan [32] obtained interesting results for the Marshall–Olkin bivariate distributions. Further, Dey et al. [33] studied reliability estimation for a Kumaraswamy distribution. Many such work have also been done in literature such as Kızılaslan and Nadar [34], Kızılaslan [35], Dey and Moala [36], Singh et al. [37], Saini et al. [38], Wang et al. [39] Akgul [40], Zhu [41], Singh et al. [42], Singh et al. [43], Kumari and Pathak [44] and Kohansal et al. [45].

In life testing experiments, it is observed that observation of failure of all the testing units is not possible or sometimes not desirable due to various associated costs such as monetary and time costs. Removal of testing units before their actual failure is widely known as censoring. Censored data has unknown values beyond a bound on either end left or right. Censoring is also categorized in terms of testing time and number of items failed. These are known as type-I and type-II censoring schemes. In the first case, the testing of units is carried out for a prefixed testing time. While under type-II censoring, a number of failures desired is prefixed, and the experiment stops when that number is reached. The major drawback of these methods is that the removal of live testing units is not allowed in between the testing period. Keeping this in mind, a censoring scheme with additional property allowing withdrawal of testing units during the testing period is introduced in literature and termed as progressive censoring. It is further categorized as type-I and type-II, progressively censoring according to the test termination mechanism. Our study in the presented paper includes a study based on progressively type-II censored (PCS) data. It is described as follows: N units are placed by an experimenter to study their life, and the number of failures, say n, is to be observed. When the first unit fails, $R_{1}$ out of the remaining units are removed randomly. Once the second unit failure is observed, random removal of $R_{2}$ surviving units is done. This process continues. Finally, after the pre-determined nth failure occurs, all surviving units are removed. The reliability estimation of multicomponent stress–strength systems is, as discussed above, considered by many researchers, but studies are also performed under censored data. Some of the articles considering censored data for the study of the MSS model can be listed as Kohansal [46], who considered the Kumaraswamy distribution under progressive censoring for statistical inferences of reliability of a multicomponent stress–strength system. Similarly, Kayal et al. [47], Maurya and Tripathi [48], Jha et al. [49], Mahto et al. [50], Mahto and Tripathi [51], Kohansal et al. [52], Singh et al. [37], Saini et al. [38], Wang et al. [39] Akgul [40], Zhu [41], Singh et al. [42], Singh et al. [43], Kumari and Pathak [44] and Kohansal et al. [45] have also considered the multicomponent stress–strength under some type of censored data.

In this paper, we consider the study of an MSS model under progressively censored data with a highly flexibility two-parameter distribution, namely logistic-exponential distribution. This distribution can exhibit constant, increasing, decreasing, and bathtub upside-down failure rate shapes, making this distribution a desirable choice for studying multicomponent systems with various failure patterns. Because of the flexibility of this distribution, it can be used as an alternative model to many of the existing studies for multicomponent systems using different distributions. Therefore, the study of reliability of MSS model under logistic exponential distribution based on progressively type-II censored data can be significantly important and a novel alternative of the existing models. The proposed model can have a wide range of applications in the fields of life testing of electronic, electrical, and mechanical equipment, civil structures such as hanging bridges, buildings with multiple pillars, dams, etc., medical life testing experiments, etc., making this study a very important and novel for such real-life applications.

The article is arranged as follows. The model description is presented in Section 2. Under the assumption that common parameter η is unknown, maximum likelihood estimation (MLE) of $R_{s,k}$ is considered in Section 3.1 and the associated asymptotic confidence interval (ACI) is obtained in Section 3.2. Further Bayesian point estimator using Lindley approximation is obtained in Section 3.3. In Section 3.4, Bayesian point and interval estimators are obtained using the MCMC technique. Next, in Section 4, all the results are also obtained for the case when η is considered to be known. In Section 5, an extensive simulation study is conducted, and then, for illustration purposes, a real data set is analyzed in Section 7. Finally, conclusions are made in Section 8.

## 2 Model description

Lan and Leemis [53] proposed and studied logistic exponential distribution whose density and distribution functions appear in a nice closed form. Statistical properties of this distribution are extensively studied, and it has been shown that this distribution is quite flexible in fitting a large class of lifetime data sets. Its flexibility stems from the fact that it exhibits constant, increasing, and decreasing bathtub and bathtub upside-down failure rate shapes. Various applications of this distribution can be found in old to new literature, such as Myers et al. [54], Chatterjee and Singh [55], van Staden and King [56], Dutta and Kayal [57], Olapade [58] and Dey et al. [59]. Recently Mahto et al. [50] considered logistic exponential distribution and obtained inferences under progressively censored progressive-stress accelerated life testing model. The probability density function (pdf) of the logistic exponential distribution is as below

(2) $$f\left(t;\lambda \;\eta \right)=\frac{\lambda \;\eta \;e^{\eta \;t}{\left(e^{\eta \;t}-1\right)}^{\lambda -1}}{{\left[1+{\left(e^{\eta \;t}-1\right)}^{\lambda }\right]}^{2}};\;t>0\;,\lambda ,\eta >0,$$

where η>0 is the scale parameter and λ>0 is the shape parameter, and, in addition to that, the cumulative distribution function (cdf) is as below

(3) $$F\left(t;\lambda \;\eta \right)=1-{\left[1+{\left(e^{\eta \;t}-1\right)}^{\lambda }\right]}^{-1}.$$

In the special case when λ=1, this distribution turns into an exponential distribution, which belongs to increasing failure rate as well as decreasing failure rate classes. The same belongs to the bathtub as well as upside-down bathtub classes for the values 0<λ<1 and λ>1, respectively. This is only a parameter distribution with such high flexibility. As discussed above, this distribution can exhibit constant, increasing, decreasing, bathtub and bathtub upside-down failure rate shapes, which makes this distribution a desirable alternative to many of the existing distributions for studying multicomponent systems.

## 3 Inference of $R_{s,k}$ when η is unknown

### 3.1 MLE

Let X∼LeD(λ,η) and Y∼LeD(α,η) be distributed as independent variables where η is the common parameter. Now, consider a progressively censored sample $\left(X_{i1},\ldots ,X_{ik}\right)$, i=1,2,…,n, from LeD(λ,η) under PCS scheme $\left(K,k,R_{1},R_{2},\ldots ,R_{k}\right)$ and in the similar fashion, let $\left(Y_{1},Y_{2},\ldots ,Y_{n}\right)$ be a progressively censored sample from LeD(α,η) under the PCS $\left(N,n,S_{1},S_{2},\ldots ,S_{n}\right)$. Under progressively censored data, the aim is to study the MSS reliability

$$R_{s,k}=P\left[\text{at least}\;s\;\text{of}\;\left(X_{1},\ldots ,X_{k}\right)\;\text{exceed}\;Y\right].$$

Then, MSS reliability for the LeD model is given by

(4) Rs,k=∑p=skkp∫0∞11+(eηy−1)λp1−11+(eηy−1)λk−pαηeηy(eηy−1)α−1[1+(eηy−1)α]2dy=∑p=sk∑j=0k−pkpk−pj(−1)jα∫0∞(1+uλ)−(p+j)αuα−1(1+uα)−2du=∑p=sk∑j=0k−pkpk−pj(−1)j∫1∞[1+(t−1)λα]−(p+j)t−2dt.

First it is required to calculate the maximum likelihood estimates of λ, α and η. Consider that n systems are put on a life-test experiment. Then the observed data are represented as follows:

$$\overset{\text{Strength Variables}}{\left(\begin{array}{cccccc} X_{11} & X_{12} & . & . & . & X_{1k}\\ X_{21} & X_{22} & . & . & . & X_{2k}\\ . & . & . & & & .\\ . & . & & . & & .\\ . & . & & & . & .\\ X_{n1} & X_{n2} & . & . & . & X_{nk} \end{array}\right)}\;\;\text{and}\;\overset{\overset{\text{Stress}}{\text{Variables}}}{\left(\begin{array}{c} Y_{1}\\ Y_{2}\\ .\\ .\\ .\\ Y_{n} \end{array}\right)}$$

where $\left(X_{i1},\ldots ,X_{ik}\right),i=1,\ldots ,n$, is a PCS sample from LeD(λ,η) with scheme $\left(K,k,R_{1},R_{2},\ldots ,R_{k}\right)$. Similarly, $(Y_{1}$, ..., $Y_{n})$ is a PCS sample under the scheme {N, n, $S_{1}$, $S_{2}$, ..., $S_{n}$}. The likelihood function of model parameters is given by

(5) $$L\left(\lambda ,\alpha ,\eta \right)=c_{1}\overset{n}{\underset{i=1}{\prod }}c_{2}\overset{k}{\underset{j=1}{\prod }}\left[f\left(x_{ij}\right)\left(1-F{\left(x_{ij}\right)}^{R_{j}}\right)\right]f\left(y_{i}\right){\left(1-F\left(y_{i}\right)\right)}^{S_{i}}$$

where,

$$c_{1}=N\left(N-S_{1}-1\right)...\left(N-S_{1}-\cdots -S_{n-1}-n+1\right),$$

$$c_{2}=k\left(k-R1-1\right)...\left(k-R_{1}-\cdots -R_{k-1}-k+1\right).$$

Then using (2), (3), we have

$$L\left(\text{data}|\lambda ,\alpha ,\eta \right)=c_{1}c_{2}^{n}\lambda^{nk}\alpha^{n}\eta^{n\left(k+1\right)}\overset{n}{\underset{i=1}{\prod }}\overset{k}{\underset{j=1}{\prod }}\frac{e^{\eta x_{ij}}{\left(e^{\eta x_{ij}}-1\right)}^{\lambda -1}}{{\left[1+{\left(e^{\eta x_{ij}}-1\right)}^{\lambda }\right]}^{R_{j}+2}}\overset{n}{\underset{i=1}{\prod }}\frac{e^{\eta y_{i}}{\left(e^{\eta y_{i}}-1\right)}^{\alpha -1}}{{\left[1+{\left(e^{\eta y_{i}}-1\right)}^{\alpha }\right]}^{S_{i}+2}}.$$

Now loglikelihood function turns out as

(6) $$l\left(\lambda ,\alpha ,\eta \right)=n\;k\text{log}\left(\lambda \right)+n\text{log}\left(\alpha \right)+n\left(k+1\right)\text{log}\left(\eta \right)+\eta \overset{n}{\underset{i=1}{\sum }}\overset{k}{\underset{j=1}{\sum }}x_{ij}+\left(\lambda -1\right)\overset{n}{\underset{i=1}{\sum }}\overset{k}{\underset{j=1}{\sum }}\text{log}\left(e^{\eta x_{ij}}-1\right)-\;\overset{n}{\underset{i=1}{\sum }}\overset{k}{\underset{j=1}{\sum }}\left(R_{j}+2\right)\text{log}\left(1+{\left(e^{\eta x_{ij}}-1\right)}^{\lambda }\right)+\eta \overset{n}{\underset{i=1}{\sum }}y_{i}+\left(\alpha -1\right)\overset{n}{\underset{i=1}{\sum }}\text{log}\left(e^{\eta y_{i}}-1\right)-\;\overset{n}{\underset{i=1}{\sum }}\left(S_{i}+2\right)\text{log}\left(1+{\left(e^{\eta y_{i}}-1\right)}^{\alpha }\right)$$

The respective MLEs $\overset{ˆ}{\lambda }$, $\overset{ˆ}{\alpha }$ and $\overset{ˆ}{\eta }$ of λ, α and η can be obtained as the solution of following equations:

(7) $$\frac{\partial l}{\partial \lambda }=\frac{nk}{\lambda }+\overset{n}{\underset{i=1}{\sum }}\overset{k}{\underset{j=1}{\sum }}\text{log}\left(e^{\eta x_{ij}}-1\right)-\overset{n}{\underset{i=1}{\sum }}\overset{k}{\underset{j=1}{\sum }}\frac{\left(R_{j}+2\right){\left(e^{\eta x_{ij}}-1\right)}^{\lambda }\text{log}\left(e^{\eta x_{ij}}-1\right)}{1+{\left(e^{\eta x_{ij}}-1\right)}^{\lambda }}=0$$

(8) $$\frac{\partial l}{\partial \alpha }=\frac{n}{\alpha }+\overset{n}{\underset{i=1}{\sum }}\text{log}\left(e^{\eta y_{i}}-1\right)-\overset{n}{\underset{i=1}{\sum }}\frac{\left(S_{i}+2\right){\left(e^{\eta y_{i}}-1\right)}^{\alpha }\text{log}\left(e^{\eta y_{i}}-1\right)}{1+{\left(e^{\eta y_{i}}-1\right)}^{\alpha }}=0$$

(9) $$\frac{\partial l}{\partial \eta }=\frac{n\left(k+1\right)}{\eta }+\overset{n}{\underset{i=1}{\sum }}\overset{k}{\underset{j=1}{\sum }}x_{ij}+\left(\lambda -1\right)\overset{n}{\underset{i=1}{\sum }}\overset{k}{\underset{j=1}{\sum }}\frac{x_{ij}e^{\eta x_{ij}}}{e^{\eta x_{ij}}-1}-\overset{n}{\underset{i=1}{\sum }}\overset{k}{\underset{j=1}{\sum }}\frac{\left(R_{j}+2\right)\lambda x_{ij}e^{\eta x_{ij}}{\left(e^{\eta x_{ij}}-1\right)}^{\lambda -1}}{1+{\left(e^{\eta x_{ij}}-1\right)}^{\lambda }}\;+\overset{n}{\underset{i=1}{\sum }}y_{i}+\left(\alpha -1\right)\overset{n}{\underset{i=1}{\sum }}\frac{y_{i}e^{\eta y_{i}}}{e^{\eta y_{i}}-1}-\overset{n}{\underset{i=1}{\sum }}\frac{\left(S_{i}+2\right)\alpha y_{i}e^{\eta y_{i}}{\left(e^{\eta y_{i}}-1\right)}^{\alpha -1}}{1+{\left(e^{\eta y_{i}}-1\right)}^{\alpha }}=0$$

The above equations are highly non-linear. Therefore, finding analytical solution is difficult and can be solved using some numerical method such as Newton–Raphson. In this regard, some of the inbuilt packages in R programming may also be useful in solving these kind of non linear system of linear equations. The next section considers the asymptotic confidence interval for reliability $R_{s,k}$.

### 3.2 Confidence interval

MLEs based interval for the reliability is discussed in this section. The limiting distribution of $\overset{ˆ}{\theta }=\left(\overset{ˆ}{\lambda },\overset{ˆ}{\alpha },\overset{ˆ}{\eta }\right)$ is derived from the observed Fisher information matrix of θ=(λ,α,η), denoted by $I\left(\theta \right)=\left[I_{ij}\right]=\left[-\frac{\partial^{2}l}{\partial \theta_{i}\partial \theta_{j}}\right],\;i,j=1,2,3$. The elements of this matrix are obtained as follows:

$$I_{11}=\frac{nk}{\lambda^{2}}+\overset{n}{\underset{i=1}{\sum }}\overset{k}{\underset{j=1}{\sum }}\frac{\left(R_{j}+2\right){\left(\text{log}\left(e^{\eta x_{ij}}-1\right)\right)}^{2}{\left(e^{\eta x_{ij}}-1\right)}^{\lambda }}{{\left[1+{\left(e^{\eta x_{ij}}-1\right)}^{\lambda }\right]}^{2}},$$

$$I_{12}=I_{21}=0,\;I_{22}=\frac{n}{\alpha^{2}}+\overset{n}{\underset{i=1}{\sum }}\frac{\left(S_{i}+2\right){\left(\text{log}\left(e^{\eta y_{i}}-1\right)\right)}^{2}{\left(e^{\eta y_{i}}-1\right)}^{\alpha }}{{\left[1+{\left(e^{\eta y_{i}}-1\right)}^{\alpha }\right]}^{2}},$$

$$I_{13}=-\overset{n}{\underset{i=1}{\sum }}\overset{k}{\underset{j=1}{\sum }}\frac{x_{ij}e^{\eta x_{ij}}}{\left(e^{\eta x_{ij}}-1\right)}+\overset{n}{\underset{i=1}{\sum }}\overset{k}{\underset{j=1}{\sum }}\frac{\left(R_{j}+2\right){\left(e^{\eta x_{ij}}-1\right)}^{\lambda -1}x_{ij}e^{\eta x_{ij}}}{1+{\left(e^{\eta x_{ij}}-1\right)}^{\lambda }}\left(1+\frac{\lambda \;\text{log}\left(e^{\eta x_{ij}}-1\right)}{1+{\left(e^{\eta x_{ij}}-1\right)}^{\lambda }}\right)=I_{31},$$

$$I_{23}=-\overset{n}{\underset{i=1}{\sum }}\frac{y_{i}e^{\eta y_{i}}}{\left(e^{\eta y_{i}}-1\right)}+\overset{n}{\underset{i=1}{\sum }}\frac{\left(S_{i}+2\right){\left(e^{\eta y_{i}}-1\right)}^{\alpha -1}y_{i}e^{\eta y_{i}}}{1+{\left(e^{\eta y_{i}}-1\right)}^{\alpha }}\left(1+\frac{\alpha \;\text{log}\left(e^{\eta y_{i}}-1\right)}{1+{\left(e^{\eta y_{i}}-1\right)}^{\alpha }}\right),$$

$$I_{33}=\frac{n\left(k+1\right)}{\eta^{2}}+\left(\lambda -1\right)\overset{n}{\underset{i=1}{\sum }}\overset{k}{\underset{j=1}{\sum }}\frac{x_{ij}^{2}e^{\eta x_{ij}}}{{\left(e^{\eta x_{ij}}-1\right)}^{2}}+\left(\alpha -1\right)\overset{n}{\underset{i=1}{\sum }}\frac{y_{i}^{2}e^{\eta y_{i}}}{{\left(e^{\eta y_{i}}-1\right)}^{2}}+\;\overset{n}{\underset{i=1}{\sum }}\overset{k}{\underset{j=1}{\sum }}\frac{\lambda \left(R_{j}+2\right)x_{ij}^{2}e^{\eta x_{ij}}{\left(e^{\eta x_{ij}}-1\right)}^{\lambda -1}}{1+{\left(e^{\eta x_{ij}}-1\right)}^{\lambda }}\left\{1+\frac{e^{\eta xij}}{e^{\eta x_{i}j}-1}\left(\lambda -\left[1+e{\left({}^{\eta x_{ij}}-1\right)}^{\lambda }\right]\right)\right\}+\;\overset{n}{\underset{i=1}{\sum }}\frac{\alpha \left(S_{i}+2\right)y_{i}^{2}e^{\eta y_{i}}{\left(e^{\eta y_{i}}-1\right)}^{\alpha -1}}{1+{\left(e^{\eta y_{i}}-1\right)}^{\alpha }}\left\{1+\frac{e^{\eta yi}}{e^{\eta y_{i}}-1}\left(\alpha -\left[1+e{\left({}^{\eta y_{i}}-1\right)}^{\alpha }\right]\right)\right\}$$

We obtain a 100(1−ɛ)% asymptotic confidence interval (ACI) for $R_{s,k}$ as ${\overset{ˆ}{R}}_{s,k}\pm Z_{ɛ/2}\sqrt{Var\left({\overset{ˆ}{R}}_{s,k}\right)}$, where ${\overset{ˆ}{R}}_{s,k}$ is the MLE of $R_{s,k}$ and variance $Var\left({\overset{ˆ}{R}}_{s,k}\right)$ is obtained following the idea as discussed in [60].

### 3.3 Bayes estimation of $R_{s,k}$

Now we carry out Bayesian estimation of the system reliability $R_{s,k}$. Therefore, in this section we treat λ,αandη as random variables. Evaluation of Bayes estimate is carried out against squared error loss function. We take independent gamma priors for λ,αandη denoted by λ∼G ($\gamma_{1},\delta_{1}$), α∼G ($\gamma_{2},\delta_{2}$) and η∼G ($\gamma_{3},\delta_{3}$), i.e. their probability density functions are given by

$$\Phi_{1}\left(\lambda \right)\propto \lambda^{\gamma_{1}-1}e^{-\delta_{1}\lambda },\;\;\lambda >0,\;\gamma_{1},\delta_{1}>0,$$

$$\Phi_{2}\left(\alpha \right)\propto \alpha^{\gamma_{2}-1}e^{-\delta_{2}\alpha },\;\;\alpha >0,\;\gamma_{2},\delta_{2}>0,$$

$$\Phi_{3}\left(\eta \right)\propto \eta^{\gamma_{3}-1}e^{-\delta_{3}\eta },\;\;\eta >0,\;\gamma_{3},\delta_{3}>0.$$

The joint posterior of λ, α and η based on observation is given by

$$\Phi \left(\lambda ,\alpha ,\eta |\text{data}\right)=\frac{L\left(data|\lambda ,\alpha ,\eta \right)\Phi_{1}\left(\lambda \right)\Phi_{2}\left(\alpha \right)\Phi_{3}\left(\eta \right)}{\int_{0}^{\infty }\int_{0}^{\infty }\int_{0}^{\infty }L\left(data|\lambda ,\alpha ,\eta \right)\Phi_{1}\left(\lambda \right)\Phi_{2}\left(\alpha \right)\Phi_{3}\left(\eta \right)d\lambda d\alpha d\eta }$$

It is observed that the Bayes estimate cannot be derived analytically. So, we use the idea of Lindley [61] as well as MCMC method to obtain the numerical approximation for Bayes estimation which are frequent approximation methods to handle such situations.

#### 3.3.1 Lindley method

Let $\psi \left(\theta \right)=R_{s,k}$, where $\theta =\left(\theta_{1},\theta_{2},\theta_{3}\right)=\left(\lambda ,\alpha ,\eta \right)$. According to Lindley’s method,

$$E\left(\psi \left(\theta \right)|\text{data}\right)=\frac{\int \psi \left(\theta \right)\exp Q\left(\theta \right)d\theta }{\int \exp Q\left(\theta \right)d\theta }$$

where Q(θ)=l(θ)+Ω(θ), l(θ) is the log likelihood function, Ω(θ) is the logarithm of the prior density of θ. Now,

$$E\left(\psi \left(\theta \right)|\text{data}\right)=\psi +\frac{1}{2}\underset{i}{\sum }\underset{j}{\sum }\left(\psi_{ij}+2\psi_{i}\Omega_{j}\right)\tau_{ij}+\frac{1}{2}\underset{i}{\sum }\underset{j}{\sum }\underset{k}{\sum }\underset{p}{\sum }l_{ijk}\tau_{ij}\tau_{kp}\psi_{p},$$

where $\theta =\left(\theta_{1},\ldots ,\theta_{m}\right),i,j,k,p=1,2,\ldots ,m$,

$$\psi_{i}=\frac{\partial \psi }{\partial \theta_{i}},\;\psi_{ij}=\frac{\partial^{2}\psi }{\partial \theta_{i}\partial \theta_{j}},\;l_{ijk}=\frac{\partial^{3}\psi }{\partial \theta_{i}\partial \theta_{j}\partial \theta_{k}},\;\Omega_{j}=\frac{\partial \Omega }{\partial \theta_{j}},$$

and $\tau_{ij}=\left(i,j\right)\text{th}$ element of the inverse of the matrix $\left[-l_{ij}\right]$, and the whole expression is evaluated at $\overset{ˆ}{\theta }$, where $\overset{ˆ}{\theta }$ is the MLE of θ. For our case, m=3 and hence

$$E\left(\psi \left(\theta \right)|\text{data}\right)=\psi +\left(\psi_{1}d_{1}+\psi_{2}d_{2}+\psi_{3}d_{3}+d_{4}+d_{5}\right)+\frac{1}{2}\left[A\left(\psi_{1}\tau_{11}+\psi_{2}\tau_{12}+\psi_{3}\tau_{13}\right)+\;B\left(\psi_{1}\tau_{21}+\psi_{2}\tau_{22}+\psi_{3}\tau_{23}\right)+C\left(\psi_{1}\tau_{31}+\psi_{2}\tau_{32}+\psi_{3}\tau_{33}\right)\right],$$

where

$$d_{i}=\Omega_{1}\tau_{i1}+\Omega_{2}\tau_{i2}+\Omega_{3}\tau_{i3},\;i=1,2,3,$$

$$d_{4}=\psi_{12}\tau_{12}+\psi_{13}\tau_{13}+\psi_{23}\tau_{23},$$

$$d_{5}=\frac{1}{2}\left(\psi_{11}\tau_{11}+\psi_{22}\tau_{22}+\psi_{33}\tau_{33}\right),$$

$$A=l_{111}\tau_{11}+2l_{121}\tau_{12}+2l_{131}\tau_{13}+2l_{231}\tau_{23}+l_{221}\tau_{22}+l_{331}\tau_{33},$$

$$B=l_{112}\tau_{11}+2l_{122}\tau_{12}+2l_{132}\tau_{13}+2l_{232}\tau_{23}+l_{222}\tau_{22}+l_{332}\tau_{33},$$

$$C=l_{113}\tau_{11}+2l_{123}\tau_{12}+2l_{133}\tau_{13}+2l_{233}\tau_{23}+l_{223}\tau_{22}+l_{333}\tau_{33}.$$

Also,

$$\Omega_{1}=\frac{\gamma_{1}-1}{\lambda }-\delta_{1},\Omega_{2}=\frac{\gamma_{2}-1}{\alpha }-\delta_{2},\Omega_{3}=\frac{\gamma_{3}-1}{\eta }-\delta_{3}.$$

ψ1=∂ψ∂λ=∑p=sk∑j=0k−pkpk−pj(−1)j+1(p+j)α∫1∞1+(t−1)λα−p−j−1(t−1)λαlog(t−1)t−2dt

ψ2=∂ψ∂α=∑p=sk∑j=0k−pkpk−pj(−1)j(p+j)λα2∫1∞1+(t−1)λα−p−j−1(t−1)λαlog(t−1)t−2dt

ψ11=∂2ψ∂λ2=∑p=sk∑j=0k−pkpk−pj(−1)j+1(p+j)α2∫1∞1+(t−1)λα−p−j−21−(p+j)(t−1)λα×(t−1)λα(log(t−1))2t−2dt

ψ12=∂2ψ∂λ∂α=∑p=sk∑j=0k−pkpk−pj(−1)j(p+j)α3∫1∞log(t−1)t−2λ1+(t−1)λα−p−j−2

$$\;\;\;\;\;\;\left(\left(\times \left(1-\left(p+j\right){\left(t-1\right)}^{\frac{\lambda }{\alpha }}\right){\left(t-1\right)}^{\frac{\lambda }{\alpha }}\log\left(t-1\right)\right)+\left(\alpha {\left(1+{\left(t-1\right)}^{\frac{\lambda }{\alpha }}\right)}^{-p-j-2}{\left(t-1\right)}^{\frac{\lambda }{\alpha }}\right)\right]dt$$

ψ22=∂2ψ∂α2=∑p=sk∑j=0k−pkpk−pj(−1)j+1(p+j)λα4∫1∞log(t−1)t−2λ1+(t−1)λα−p−j−2

$$\;\;\;\;\;\;\left(\left(\times \left(1-\left(p+j\right){\left(t-1\right)}^{\frac{\lambda }{\alpha }}\right){\left(t-1\right)}^{\frac{\lambda }{\alpha }}\log\left(t-1\right)\right)+\left(2\alpha {\left(1+{\left(t-1\right)}^{\frac{\lambda }{\alpha }}\right)}^{-p-j-1}{\left(t-1\right)}^{\frac{\lambda }{\alpha }}\right)\right]dt$$

$$\psi_{3}=\frac{\partial R_{s,k}}{\partial \eta }=0,\text{also}\;\psi_{13}=\psi_{23}=\psi_{33}=0.$$

We get

$$\text{We get}\tau_{ij},i,j=1,2,3\;\text{from}\;l_{ij},i,j=1,2,3\;\text{and}$$

$$l_{11}=-\frac{nk}{\lambda^{2}}-\overset{n}{\underset{i=1}{\sum }}\overset{k}{\underset{j=1}{\sum }}\frac{\left(R_{j}+2\right){\left(\text{log}\left(e^{\eta x_{ij}}-1\right)\right)}^{2}{\left(e^{\eta x_{ij}}-1\right)}^{\lambda }}{{\left[1+{\left(e^{\eta x_{ij}}-1\right)}^{\lambda }\right]}^{2}}$$

$$l_{12}=l_{21}=0,\;I_{22}=-\frac{n}{\alpha^{2}}-\overset{n}{\underset{i=1}{\sum }}\frac{\left(S_{i}+2\right){\left(\text{log}\left(e^{\eta y_{i}}-1\right)\right)}^{2}{\left(e^{\eta y_{i}}-1\right)}^{\alpha }}{{\left[1+{\left(e^{\eta y_{i}}-1\right)}^{\alpha }\right]}^{2}}$$

$$l_{13}=\overset{n}{\underset{i=1}{\sum }}\overset{k}{\underset{j=1}{\sum }}\frac{x_{ij}e^{\eta x_{ij}}}{\left(e^{\eta x_{ij}}-1\right)}-\overset{n}{\underset{i=1}{\sum }}\overset{k}{\underset{j=1}{\sum }}\frac{\left(R_{j}+2\right){\left(e^{\eta x_{ij}}-1\right)}^{\lambda -1}x_{ij}e^{\eta x_{ij}}}{1+{\left(e^{\eta x_{ij}}-1\right)}^{\lambda }}\left(1+\frac{\lambda \;\text{log}\left(e^{\eta x_{ij}}-1\right)}{1+{\left(e^{\eta x_{ij}}-1\right)}^{\lambda }}\right)=l_{31}$$

$$l_{23}=\overset{n}{\underset{i=1}{\sum }}\frac{y_{i}e^{\eta y_{i}}}{\left(e^{\eta y_{i}}-1\right)}-\overset{n}{\underset{i=1}{\sum }}\frac{\left(S_{i}+2\right){\left(e^{\eta y_{i}}-1\right)}^{\alpha -1}y_{i}e^{\eta y_{i}}}{1+{\left(e^{\eta y_{i}}-1\right)}^{\alpha }}\left(1+\frac{\alpha \;\text{log}\left(e^{\eta y_{i}}-1\right)}{1+{\left(e^{\eta y_{i}}-1\right)}^{\alpha }}\right)=l_{32}$$

$$l_{33}=-\frac{n\left(k+1\right)}{\eta^{2}}-\left(\lambda -1\right)\overset{n}{\underset{i=1}{\sum }}\overset{k}{\underset{j=1}{\sum }}\frac{x_{ij}^{2}e^{\eta x_{ij}}}{{\left(e^{\eta x_{ij}}-1\right)}^{2}}-\left(\alpha -1\right)\overset{n}{\underset{i=1}{\sum }}\frac{y_{i}^{2}e^{\eta y_{i}}}{{\left(e^{\eta y_{i}}-1\right)}^{2}}\;-\overset{n}{\underset{i=1}{\sum }}\overset{k}{\underset{j=1}{\sum }}\frac{\lambda \left(R_{j}+2\right)x_{ij}^{2}e^{\eta x_{ij}}{\left(e^{\eta x_{ij}}-1\right)}^{\lambda -1}}{1+{\left(e^{\eta x_{ij}}-1\right)}^{\lambda }}\left\{1+\frac{e^{\eta xij}\left(\lambda -\left[1+e{\left({}^{\eta x_{ij}}-1\right)}^{\lambda }\right]\right)}{e^{\eta x_{i}j}-1}\right\}\;-\overset{n}{\underset{i=1}{\sum }}\frac{\alpha \left(S_{i}+2\right)y_{i}^{2}e^{\eta y_{i}}{\left(e^{\eta y_{i}}-1\right)}^{\alpha -1}}{1+{\left(e^{\eta y_{i}}-1\right)}^{\alpha }}\left\{1+\frac{e^{\eta yi}\left(\alpha -\left[1+e{\left({}^{\eta y_{i}}-1\right)}^{\alpha }\right]\right)}{e^{\eta y_{i}}-1}\right\}$$

The third derivatives are given as follows:

$$l_{111}=\frac{2nk}{\lambda^{3}}-\overset{n}{\underset{i=1}{\sum }}\overset{k}{\underset{j=1}{\sum }}\frac{\left(R_{j}+2\right){\left(\log\left(e^{\eta x_{ij}}-1\right)\right)}^{3}{\left(e^{\eta x_{ij}}-1\right)}^{\lambda }\left[1-{\left(e^{\eta x_{ij}}-1\right)}^{\lambda }\right]}{{\left[1+{\left(e^{\eta x_{ij}}-1\right)}^{\lambda }\right]}^{3}}$$

$$l_{222}=\frac{2n}{\alpha^{3}}-\overset{n}{\underset{i=1}{\sum }}\frac{\left(S_{i}+2\right){\left(\log\left(e^{\eta y_{i}}-1\right)\right)}^{3}{\left(e^{\eta y_{i}}-1\right)}^{\alpha }\left[1-{\left(e^{\eta y_{i}}-1\right)}^{\alpha }\right]}{{\left[1+{\left(e^{\eta y_{i}}-1\right)}^{\alpha }\right]}^{3}}$$

$$l_{123}=l_{132}=l_{213}=l_{231}=l_{312}=l_{321}=l_{112}=l_{121}=l_{211}=l_{221}=l_{212}=l_{122}=0$$

$$l_{113}=-\overset{n}{\underset{i=1}{\sum }}\overset{k}{\underset{j=1}{\sum }}\frac{\left(R_{j}+2\right)x_{ij}e^{\eta x_{ij}}{\left(e^{\eta x_{ij}}-1\right)}^{\lambda -1}\log\left(e^{\eta x_{ij}}-1\right)}{{\left[1+{\left(e^{\eta x_{ij}}-1\right)}^{\lambda }\right]}^{2}}\left[2+\lambda \log\left(e^{\eta x_{ij}}-1\right)\right)\;-\left(\frac{2\lambda {\left(e^{\eta x_{ij}}-1\right)}^{\lambda }\log\left(e^{\eta x_{ij}}-1\right)}{\left[1+{\left(e^{\eta x_{ij}}-1\right)}^{\lambda }\right]}\right]$$

$$l_{223}=-\overset{n}{\underset{i=1}{\sum }}\frac{\left(S_{i}+2\right)y_{i}e^{\eta y_{i}}{\left(e^{\eta y_{i}}-1\right)}^{\alpha -1}\log\left(e^{\eta y_{i}}-1\right)}{{\left[1+{\left(e^{\eta y_{i}}-1\right)}^{\alpha }\right]}^{2}}\left[2+\alpha \log\left(e^{\eta y_{i}}-1\right)\right)\;-\left(\frac{2\alpha {\left(e^{\eta y_{i}}-1\right)}^{\alpha }\log\left(e^{\eta y_{i}}-1\right)}{\left[1+{\left(e^{\eta y_{i}}-1\right)}^{\alpha }\right]}\right]$$

$$l_{331}=-\overset{n}{\underset{i=1}{\sum }}\overset{k}{\underset{j=1}{\sum }}\frac{x_{ij}^{2}e^{\eta x_{ij}}}{{\left(e^{\eta x_{ij}}-1\right)}^{2}}-\overset{n}{\underset{i=1}{\sum }}\overset{k}{\underset{j=1}{\sum }}\frac{\left(R_{j}+2\right)x_{ij}^{2}e^{\eta x_{ij}}{\left(e^{\eta x_{ij}}-1\right)}^{\lambda -2}}{{\left[1+{\left(e^{\eta x_{ij}}-1\right)}^{\lambda }\right]}^{2}}\left[\left(\lambda e^{\eta x_{ij}}-{\left(e^{\eta x_{ij}}-1\right)}^{\lambda }-1\right)\right)$$

$$\;\;\times \left(\left(1+\lambda \log\left(e^{\eta x_{ij}}-1\right)-\frac{2\lambda {\left(e^{\eta x_{ij}}-1\right)}^{\lambda }\log\left(e^{\eta x_{ij}}-1\right)}{\left[1+{\left(e^{\eta x_{ij}}-1\right)}^{\lambda }\right]}\right)+\lambda \left(e^{\eta x_{ij}}-{\left(e^{\eta x_{ij}}-1\right)}^{\lambda }\right)\right)\;\;\times \left(\left(\log\left(e^{\eta x_{ij}}-1\right)\right)\right]$$

$$l_{332}=-\overset{n}{\underset{i=1}{\sum }}\frac{y_{i}^{2}e^{\eta y_{i}}}{{\left(e^{\eta y_{i}}-1\right)}^{2}}-\overset{n}{\underset{i=1}{\sum }}\frac{\left(S_{i}+2\right)y_{i}^{2}e^{\eta y_{i}}{\left(e^{\eta y_{i}}-1\right)}^{\alpha -2}}{{\left[1+{\left(e^{\eta y_{i}}-1\right)}^{\alpha }\right]}^{2}}\left[\left(\alpha e^{\eta y_{i}}-{\left(e^{\eta y_{i}}-1\right)}^{\alpha }-1\right)\right)$$

$$\;\;\times \left(\left(1+\alpha \log\left(e^{\eta y_{i}}-1\right)-\frac{2\alpha {\left(e^{\eta y_{i}}-1\right)}^{\alpha }\log\left(e^{\eta y_{i}}-1\right)}{\left[1+{\left(e^{\eta y_{i}}-1\right)}^{\alpha }\right]}\right)+\alpha \left(e^{\eta y_{i}}-{\left(e^{\eta y_{i}}-1\right)}^{\alpha }\log\left(e^{\eta y_{i}}-1\right)\right)\right]$$

$$l_{333}=\frac{2n\left(k+1\right)}{\eta^{3}}+\left(\lambda -1\right)\overset{n}{\underset{i=1}{\sum }}\overset{k}{\underset{j=1}{\sum }}\frac{x_{ij}^{3}e^{\eta x_{ij}}\left(e^{\eta x_{ij}}+1\right)}{{\left(e^{\eta x_{ij}}-1\right)}^{3}}+\left(\alpha -1\right)\overset{n}{\underset{i=1}{\sum }}\frac{y_{i}^{3}e^{\eta y_{i}}\left(e^{\eta y_{i}}+1\right)}{{\left(e^{\eta y_{i}}-1\right)}^{3}}-\overset{n}{\underset{i=1}{\sum }}\overset{k}{\underset{j=1}{\sum }}\left(R_{j}+2\right)\times \;\frac{\lambda x_{ij}^{2}e^{\eta x_{ij}}{\left(e^{\eta x_{ij}}-1\right)}^{\lambda -3}}{{\left[1+{\left(e^{\eta x_{ij}}-1\right)}^{\lambda }\right]}^{2}}\left[\left(\lambda e^{\eta x_{ij}}-{\left(e^{\eta x_{ij}}-1\right)}^{\lambda }-1\right)\left(x_{ij}\left(e^{\eta x_{ij}}-1\right)+\left(\lambda -2\right)x_{ij}e^{\eta x_{ij}}\right)\right)-\;\left(\left(\frac{2\lambda x_{ij}e^{\eta x_{ij}}{\left(e^{\eta x_{ij}}-1\right)}^{\lambda }}{\left[1+{\left(e^{\eta x_{ij}}-1\right)}^{\lambda }\right]}\right)+\left(\lambda x_{ij}e^{\eta x_{ij}}-\lambda x_{ij}e^{\eta x_{ij}}{\left(e^{\eta x_{ij}}-1\right)}^{\lambda -1}\right)\left(e^{\eta x_{ij}}-1\right)\right]-\;\overset{n}{\underset{i=1}{\sum }}\frac{\left(S_{i}+2\right)\alpha y_{i}^{2}e^{\eta y_{i}}{\left(e^{\eta y_{i}}-1\right)}^{\alpha -3}}{{\left[1+{\left(e^{\eta y_{i}}-1\right)}^{\alpha }\right]}^{2}}\left[\left(\alpha e^{\eta y_{i}}-{\left(e^{\eta y_{i}}-1\right)}^{\alpha }-1\right)\left(y_{i}\left(e^{\eta y_{i}}-1\right)+\left(\alpha -2\right)y_{i}e^{\eta y_{i}}\right)\right)-\;\left(\left(\frac{2\alpha y_{i}e^{\eta y_{i}}{\left(e^{\eta y_{i}}-1\right)}^{\alpha }}{\left[1+{\left(e^{\eta y_{i}}-1\right)}^{\alpha }\right]}\right)+\left(\alpha y_{i}e^{\eta y_{i}}-\alpha y_{i}e^{\eta y_{i}}{\left(e^{\eta y_{i}}-1\right)}^{\alpha -1}\right)\left(e^{\eta y_{i}}-1\right)\right].$$

So,

$$d_{4}=\psi_{12}\tau_{12},\;d_{5}=\frac{1}{2}\left(\psi_{11}\tau_{11}+\psi_{22}\tau_{22}\right),$$

$$A=l_{111}\tau_{11}+2l_{131}\tau_{13}+l_{331}\tau_{33},$$

$$B=2l_{232}\tau_{23}+l_{222}\tau_{22}+l_{332}\tau_{33},$$

$$C=l_{113}\tau_{11}+2l_{133}\tau_{13}+2l_{233}\tau_{23}+l_{223}\tau_{22}+l_{333}\tau_{33}.$$

Therefore, the Bayes estimator of $R_{s,k}$ using Lindley’s approximation is given by:

$${\overset{ˆ}{R}}_{s,k}^{Lin}=R_{s,k}+\left(\psi_{1}d_{1}+\psi_{2}d_{2}+d_{4}+d_{5}\right)+\frac{1}{2}\left[A\left(\psi_{1}\tau_{11}+\psi_{2}\tau_{12}\right)+B\left(\psi_{1}\tau_{21}+\psi_{2}\tau_{22}\right)\;+C\left(\psi_{1}\tau_{31}+\psi_{2}\tau_{32}\right)\right].$$

The Lindley’s method provides approximate point estimates for the model parameters and the associated reliability function but this method is unable to find the Bayesian interval estimates. Therefore, some other method is needed to fulfill the shortcoming of this method. Thus MCMC method is proposed further to generate samples for finding associated Bayesian interval estimates the reliability function $R_{s,k}$.

### 3.4 MCMC method

The marginal posterior distributions for the three parameters λ, α and η are obtained as

(10) $$\Phi \left(\lambda |\alpha ,\eta ,\text{data}\right)\propto \lambda^{nk+\gamma_{1}-1}\overset{n}{\underset{i=1}{\prod }}\overset{k}{\underset{j=1}{\prod }}\frac{{\left(e^{\eta x_{ij}}-1\right)}^{\lambda -1}}{{\left[1+{\left(e^{\eta x_{ij}}-1\right)}^{\lambda }\right]}^{R_{j}+2}}$$

(11) $$\Phi \left(\alpha |\lambda ,\eta ,\text{data}\right)\propto \alpha^{n+\gamma_{2}-1}\overset{n}{\underset{i=1}{\prod }}\frac{{\left(e^{\eta y_{i}}-1\right)}^{\alpha -1}}{{\left[1+{\left(e^{\eta y_{i}}-1\right)}^{\alpha }\right]}^{S_{i}+2}}$$

(12) $$\Phi \left(\eta |\lambda ,\alpha ,\text{data}\right)\propto \eta^{\left(n\left(k+1\right)+\gamma_{3}-1\right)}e^{-\delta_{3}\eta }\overset{n}{\underset{i=1}{\prod }}\overset{k}{\underset{j=1}{\prod }}\frac{e^{\eta x_{ij}}{\left(e^{\eta x_{ij}}-1\right)}^{\lambda -1}}{{\left[1+{\left(e^{\eta x_{ij}}-1\right)}^{\lambda }\right]}^{R_{j}+2}}\overset{n}{\underset{i=1}{\prod }}\frac{e^{\eta y_{i}}{\left(e^{\eta y_{i}}-1\right)}^{\alpha -1}}{{\left[1+{\left(e^{\eta y_{i}}-1\right)}^{\alpha }\right]}^{S_{i}+2}}$$

**Fig. 1:**
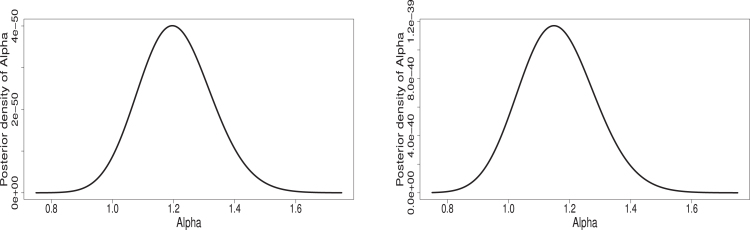
Plots for Φ(λ∣α,η,data) under censoring schemes $\left(R_{1},S_{1}\right)$(left) and $\left(R_{2},S_{1}\right)$(right).

**Fig. 2:**
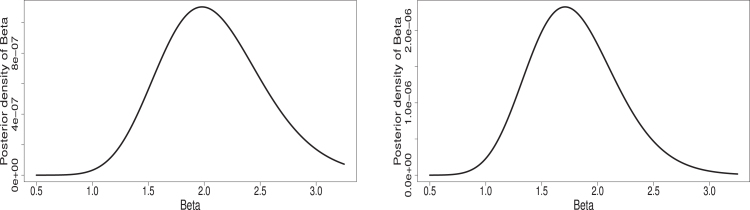
Plots for Φ(α∣λ,η,data) under censoring schemes $\left(R_{1},S_{1}\right)$(left) and $\left(R_{2},S_{1}\right)$(right).

**Fig. 3:**
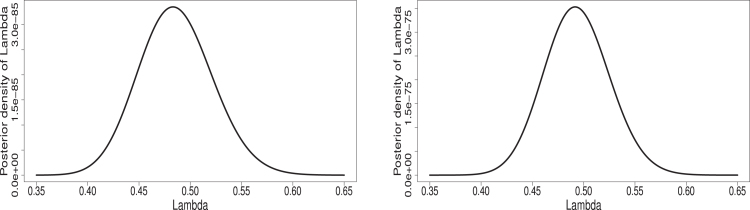
Plots for Φ(η∣λ,α,data) under censoring schemes $\left(R_{1},S_{1}\right)$(left) and $\left(R_{2},S_{1}\right)$(right).

It is observed that finding the above conditional posterior distributions in the form of some known distribution is difficult. Therefore, direct generation of samples for the parameters λ, α and η and associated reliability function $R_{s,k}$ is not possible. But the graphical plots of the conditional posterior distributions show that these are unimodal and roughly symmetric (see, Fig. 1, Fig. 2, Fig. 3). One way to handle such situation is to generate samples from proposal distribution as normal distribution and in this regard, one may refer to Ahmed [62] for detailed discussion. Therefore, Metropolis-Hastings (MH) algorithm which used normal proposal distributions for the parameters, is used to generate desired posterior samples for $R_{s,k}$. A step by step algorithm is presented below.

Step 1: Initialize (λ,α,η) as $\left(\lambda^{0},\alpha^{0},\eta^{0}\right)$.
Step 2: Set count i=1.
Step 3: Generate the random sample for λ, α and η as
  $$\lambda ∼Nor\left(\lambda^{i},\tau_{11}\right),\;\;\;\alpha ∼Nor\left(\alpha^{i},\tau_{22}\right)\;\text{and}\;\;\;\eta ∼Nor\left(\eta^{i},\tau_{33}\right),$$
  where $\tau_{ij}$ denotes the (i,j)th element of the variance–covariance matrix τ evaluated at $\left(\lambda^{i-1},\alpha^{i-1},\eta^{i-1}\right)$.
Step 4: Evaluate the ratio
  $$P=\frac{\Phi \left(\lambda^{i},\alpha^{i},\eta^{i}|\text{data}\right)}{\Phi \left(\lambda^{i},\alpha^{i},\eta^{i}|\text{data}\right)}.$$
Step 5: Accept $\left(\lambda^{i},\alpha^{i},\eta^{i}\right)$ with probability min(1,P).
Step 6: Evaluate ${\overset{ˆ}{R}}_{s,k}^{i}$ at $\left(\lambda^{i},\alpha^{i},\eta^{i}\right)$
Step 7: Repeat steps (3)–(6), N number of times to obtain N random samples $R_{s,k}^{i}$.

To remove the dependency on the initial guess, we remove $N^{\prime }$ initial samples which is known as burn-in period where the chains are considered to be in transient phase. Suppose $N^{*}=N-N^{\prime }$, then the reliability estimate ${\overset{ˆ}{R}}_{s,k}$ under the squared error loss can be obtained as

$${\overset{ˆ}{R}}_{s,k}=\frac{1}{N^{*}}\overset{N^{*}}{\underset{\zeta =1}{\sum }}{\overset{ˆ}{R}}_{s,k}^{\zeta }.$$

Further, the credible intervals are also constructed using the generated posterior samples following the idea introduced by Chen and Shao [63]. For this, we order $R_{s,k}^{i}$ for i=1,2,…,N, like $R_{s,k}^{\left(1\right)},R_{s,k}^{\left(2\right)},\ldots ,R_{s,k}^{\left(N\right)}$. Then, by removing equal number of samples from both lower and upper sides, 100(1−ξ)% credible interval is given by $\left(R_{s,k}^{i_{1}},R_{s,k}^{i_{2}}\right)$, where, $i_{1}$ and $i_{2}$ are such that $i_{1},i_{2}\in \left\{1,2,\ldots ,N\right\},\;i_{1}<i_{2},\;\;R_{s,k}^{i_{1}}\leq 1-\xi \leq R_{s,k}^{i_{2}}$.

## 4 Inference of $R_{s,k}$ when η is known

### 4.1 MLE

Here MLE of MSS reliability is evaluated under PCS data. Since η is known, we can obtain MLEs of shape parameters λ and α by solving Eqs. (7), (8). This system of equations can again be solved using non-linear methods like Newton or Broyden. After that, these estimates can be substituted in Eq. (4) to get the desired MLE of $R_{s,k}$.

### 4.2 Confidence interval estimation

To obtain the asymptotic confidence distribution of $\overset{ˆ}{\theta }=\left(\overset{ˆ}{\lambda },\overset{ˆ}{\alpha }\right)$, we refer back to Section 3.2 and notice that since η is known, $I_{13}=I_{31}=I_{23}=I_{32}=I_{33}=0$, and the Fisher’s Information matrix is of the size 2 × 2. After that, the asymptotic confidence interval can be found in a similar manner by using the variance–covariance matrix $I^{-1}\left(\theta \right)$.

### 4.3 Bayesian estimation

Similar to Section 3.3, we carry out the Bayesian estimation of $R_{s,k}\;$ when η is known. further parameters λ and α are unknown. We assume as previously that λ and α have independent gamma priors denoted by λ∼G ($\gamma_{1},\delta_{1}$) and α∼G ($\gamma_{2},\delta_{2}$) i.e there PDFs are given by

$$\Phi_{1}\left(\lambda \right)\propto \lambda^{\gamma_{1}-1}e^{-\delta_{1}\lambda },\;\;\lambda >0,\;\gamma_{1},\delta_{1}>0,$$

$$\Phi_{2}\left(\alpha \right)\propto \alpha^{\gamma_{2}-1}e^{-\delta_{2}\alpha },\;\;\alpha >0,\;\gamma_{2},\delta_{2}>0.$$

Therefore joint posterior of λ, α based on the observed sample is given by

$$\Phi \left(\lambda ,\alpha ,\eta |\text{data}\right)=\frac{L\left(data|\lambda ,\alpha ,\eta \right)\Phi_{1}\left(\lambda \right)\Phi_{2}\left(\alpha \right)\Phi_{3}\left(\eta \right)}{\int_{0}^{\infty }\int_{0}^{\infty }\int_{0}^{\infty }L\left(data|\lambda ,\alpha ,\eta \right)\Phi_{1}\left(\lambda \right)\Phi_{2}\left(\alpha \right)\Phi_{3}\left(\eta \right)d\lambda d\alpha d\eta }$$

It is clear that the Bayes estimate can again not be derived analytically. So, we use the idea of Lindley [61] as well as MCMC method to obtain the numerical approximation for our Bayes estimates.

#### 4.3.1 Lindley’s approximation

Let $\psi \left(\theta \right)=R_{s,k}$, where $\theta =\left(\theta_{1},\theta_{2}\right)=\left(\lambda ,\alpha \right)$. Similar to Section 4.3.1,

$$E\left(\psi \left(\theta \right)|\text{data}\right)=\psi +\left(\psi_{1}p_{1}+\psi_{2}p_{2}+p_{3}\right)+\frac{1}{2}\left[P\left(\psi_{1}\tau_{11}+\psi_{2}\tau_{12}\right)+Q\left(\psi_{1}\tau_{21}+\psi_{2}\tau_{22}\right)\right],$$

where

$$p_{i}=\Omega_{1}\tau_{i1}+\Omega_{2}\tau_{i2},\;i=1,2,$$

$$p_{3}=\frac{1}{2}\left(\psi_{11}\tau_{11}+\psi_{12}\tau_{12}+\psi_{21}\tau_{21}+\psi_{22}\tau_{22}\right),$$

$$P=l_{111}\tau_{11}+l_{121}\tau_{12}+l_{211}\tau_{21}+l_{221}\tau_{22},$$

$$Q=l_{112}\tau_{11}+l_{122}\tau_{12}+l_{212}\tau_{21}+l_{222}\tau_{22}.$$

Other expressions can be found in Section 4.3.1. Hence, $P=l_{111}\tau_{11}$ and $Q=l_{222}\tau_{22}$. Thus desired estimator of $R_{s,k}$ as given by Lindley’s approximation is :

$${\overset{ˆ}{R}}_{s,k}^{Lin}=R_{s,k}+\left(\psi_{1}p_{1}+\psi_{2}p_{2}+p_{3}\right)+\frac{1}{2}\left[P\left(\psi_{1}\tau_{11}+\psi_{2}\tau_{12}\right)+Q\left(\psi_{1}\tau_{21}+\psi_{2}\tau_{22}\right)\right].$$

All the parameters are evaluated at $\left(\overset{ˆ}{\lambda },\overset{ˆ}{\alpha }\right)$.

### 4.4 MCMC method

With a given η, posterior distributions for λ and α can be obtained as follows:

(13) $$\Phi \left(\lambda |\alpha ,\eta ,\text{data}\right)\propto \lambda^{nk+\gamma_{1}-1}\overset{n}{\underset{i=1}{\prod }}\overset{k}{\underset{j=1}{\prod }}\frac{{\left(e^{\eta x_{ij}}-1\right)}^{\lambda -1}}{{\left[1+{\left(e^{\eta x_{ij}}-1\right)}^{\lambda }\right]}^{R_{j}+2}}$$

and

(14) $$\Phi \left(\alpha |\lambda ,\eta ,\text{data}\right)\propto \alpha^{n+\gamma_{2}-1}\overset{n}{\underset{i=1}{\prod }}\frac{{\left(e^{\eta y_{i}}-1\right)}^{\alpha -1}}{{\left[1+{\left(e^{\eta y_{i}}-1\right)}^{\alpha }\right]}^{S_{i}+2}}.$$

Similar to the case when η is unknown, we cannot find posterior distributions in the form of some known distribution. But the graphical plots suggest that these are unimodal and roughly symmetric, so we generate the posterior samples for $R_{s,k}$ using normal proposal distribution. The sample can be generated following the steps explained below:

Step 1: Initialize (λ,α) as $\left(\lambda^{0},\alpha^{0}\right)$.
Step 2: Set count i=1.
Step 3: Generate the random sample for λ and α as
  $$\lambda ∼Nor\left(\lambda^{i},\tau_{11}\right)\;\text{and}\;\alpha ∼Nor\left(\alpha^{i},\tau_{22}\right),$$
  where $\tau_{ij}$ denotes the (i,j)th element of the variance–covariance matrix τ evaluated at $\left(\lambda^{i-1},\alpha^{i-1}\right)$.
Step 4: Evaluate the ratio
  $$P=\frac{\Phi \left(\lambda^{i},\alpha^{i}|\text{data}\right)}{\Phi \left(\lambda^{i},\alpha^{i}|\text{data}\right)}.$$
Step 5: Accept $\left(\lambda^{i},\alpha^{i}\right)$ with probability min(1,P).
Step 6: Evaluate ${\overset{ˆ}{R}}_{s,k}^{i}$ at $\left(\lambda^{i},\alpha^{i}\right)$
Step 7: Repeat steps (3)–(6), N number of times to obtain N random samples $R_{s,k}^{i}$.

In similar manner to the case with unknown η, after removing $N^{\prime }$ burn-in samples. We obtain the reliability estimate ${\overset{ˆ}{R}}_{s,k}$ as

$${\overset{ˆ}{R}}_{s,k}=\frac{1}{N^{*}}\overset{N^{*}}{\underset{\zeta =1}{\sum }}{\overset{ˆ}{R}}_{s,k}^{\zeta },$$

where $N^{*}=N-N^{\prime }$. Further, credible intervals are constructed using similar idea as discussed earlier.

## 5 Simulation

We have considered an extensive simulation study to access the behavior of various methods discussed. For the case when η is unknown, the study is based on two sets of parameter values $\theta_{1}=\left(1.2,2,0.5\right)$ and $\theta_{2}=\left(0.5,2.25,0.75\right)$. For known η, sets of parameter values are set as $\theta_{3}=\left(1.2,2\right)$ and $\theta_{4}=\left(0.75,2.5\right)$ and known value of η is set as η=1.25 which acts as a constant through out the study. The values of (N,n) and (K,k) are set as (15,10) and (10,6) respectively. The classical as well as Bayesian estimates for the MSS reliability $R_{s,k}$ is considered under various combinations of censoring schemes $\left(R_{i},S_{i}\right),\;i=1,2,3$. The considered schemes are listed in Table 1.

For the Bayesian estimation, priors for $\theta_{1}$ are set as $\left(\gamma_{1},\delta_{1}\right)=\left(2,1.67\right)$, $\left(\gamma_{2},\delta_{2}\right)=\left(3,1.5\right)$ and $\left(\gamma_{3},\delta_{3}\right)=\left(3,6\right)$, for $\theta_{2}$, are set as $\left(\gamma_{1},\delta_{1}\right)=\left(3,6\right)$, $\left(\gamma_{2},\delta_{2}\right)=\left(4,2\right)$ and $\left(\gamma_{3},\delta_{3}\right)=\left(3,4\right)$, for $\theta_{3}$, are set as $\left(\gamma_{1},\delta_{1}\right)=\left(2,1.67\right)$, $\left(\gamma_{2},\delta_{2}\right)=\left(3,1.5\right)$ and similarly, for $\theta_{4}$, are set as $\left(\gamma_{1},\delta_{1}\right)=\left(4,4\right)$, $\left(\gamma_{2},\delta_{2}\right)=\left(5,2\right)$. The estimates are obtained for an 2-out-of-6 MSS system, that is, $R_{2,6}$ and also for an 3-out-of-6 MSS system, that is, $R_{3,6}$. The classical point estimates are obtained using the maximum likelihood estimation method and the Bayesian point estimates are obtained from Lindley and MCMC methods. The asymptotic intervals are constructed using asymptotic normality property of MLE. Note that Lindley’s method is unable to construct credible intervals, so these are constructed using MCMC data. The confidence level is taken to be as 95%. Keeping an eye on the effect of prior choices all the Bayesian estimates are evaluated against informative as well as non-informative priors. The various point estimates are compared under the standard MSE and bias criteria. Furthermore, comparison of intervals are done in terms of their average interval length (AIL) and coverage probabilities (CPs). All the results of classical and Bayesian estimates of reliability $R_{s,k}$ with corresponding MSEs for the case when η is unknown are tabulated in Table 2 and for the case when η is known, the results are tabulated in Table 3. In the similar fashion, the interval estimates of reliability $R_{s,k}$ with AIL and CPs for ACI and Bayesian methods with informative and non-informative priors for the case when η is unknown are tabulated in Table 4 and the results for the case when η is known are tabulated in Table 5. It is to note that to see the consistency in the performance of the proposed model, we have obtained the point and interval estimates of $R_{s,k}$ for four sets of parameters with varying censoring schemes and for MCMC sample generation, we have removed 1000 initial samples as burn in samples. All the computations in this manuscript are conducted using R programming software in an Intel Core i7 processor. We suggest the readers to write to corresponding author to get the codes for reproducing any/all of the results presented.

**Table 1:** Censoring schemes.

(k,K)		CS	(n,N)		CS
(10,6)	R1	(4,0,0,0,0,0)	(15,10)	S1	(5,0,0,0,0,0,0,0,0,0)
	R2	(0,0,0,0,0,4)		S2	(0,0,0,0,0,0,0,0,0,5)
	R3	(1,1,1,1,0,0)		S3	(1,0,1,0,1,0,1,0,1,0)

**Table 2:** Classical and Bayesian point estimates and associated MSEs of $R_{s,k}$ when η is unknown.

$\theta_{i}$	s	$R_{s,k}$	CS		MLE			Informative prior					Non-informative prior			
					${\overset{ˆ}{R}}_{s,k}$	MSE		Lindley		MCMC			Lindley		MCMC	
								${\overset{ˆ}{R}}_{s,k}$	MSE	${\overset{ˆ}{R}}_{s,k}$	MSE		${\overset{ˆ}{R}}_{s,k}$	MSE	${\overset{ˆ}{R}}_{s,k}$	MSE
$\theta_{1}$	2	0.783320	$R_{1}S_{1}$		0.784801	0.001027		0.782967	0.000498	0.779182	0.000757		0.783952	0.000784	0.778780	0.001094
			$R_{1}S_{2}$		0.787458	0.001234		0.782490	0.000500	0.778549	0.000903		0.784675	0.000881	0.778819	0.001260
			$R_{1}S_{3}$		0.786702	0.001201		0.783113	0.000530	0.779149	0.000895		0.785128	0.000803	0.780161	0.001167
			$R_{2}S_{1}$		0.783647	0.001267		0.783077	0.000591	0.778271	0.000980		0.784513	0.000836	0.778907	0.001201
			$R_{2}S_{2}$		0.785728	0.001402		0.782484	0.000575	0.778046	0.001014		0.785825	0.000902	0.780519	0.001302
			$R_{2}S_{3}$		0.784831	0.001307		0.782758	0.000583	0.778367	0.000980		0.784353	0.000784	0.779068	0.001146
			$R_{3}S_{1}$		0.784147	0.001240		0.782981	0.000585	0.778434	0.000960		0.782617	0.000780	0.776694	0.001122
			$R_{3}S_{2}$		0.786272	0.001381		0.782356	0.000572	0.778200	0.000992		0.784449	0.000885	0.779053	0.001262
			$R_{3}S_{3}$		0.785354	0.001284		0.782644	0.000571	0.778403	0.000964		0.784151	0.000816	0.778650	0.001169
	3	0.599772	$R_{1}S_{1}$		0.600743	0.000217		0.601343	0.000103	0.598529	0.000160		0.601976	0.000149	0.598636	0.000201
			$R_{1}S_{2}$		0.601972	0.000244		0.601213	0.000009	0.598328	0.000165		0.602452	0.000181	0.598739	0.000247
			$R_{1}S_{3}$		0.601904	0.000243		0.601566	0.000107	0.598972	0.000170		0.60213	0.000160	0.598870	0.000215
			$R_{2}S_{1}$		0.600797	0.000232		0.601559	0.000107	0.598647	0.000165		0.601634	0.000159	0.598146	0.000204
			$R_{2}S_{2}$		0.601873	0.000268		0.601441	0.000106	0.598614	0.000177		0.602371	0.000177	0.598616	0.000241
			$R_{2}S_{3}$		0.601385	0.000249		0.601446	0.000110	0.598654	0.000169		0.000241	0.000166	0.599164	0.000214
			$R_{3}S_{1}$		0.600821	0.000222		0.601452	0.000103	0.598424	0.000157		0.601298	0.000157	0.597741	0.000211
			$R_{3}S_{2}$		0.602149	0.000263		0.601408	0.000103	0.598690	0.000173		0.602624	0.000184	0.599207	0.000241
			$R_{3}S_{3}$		0.601654	0.000243		0.601424	0.000107	0.598745	0.000165		0.601970	0.000162	0.598677	0.000219
$\theta_{2}$	2	0.865948	$R_{1}S_{1}$		0.865535	0.000154		0.859662	0.000149	0.861861	0.000143		0.859457	0.000234	0.860732	0.000213
			$R_{1}S_{2}$		0.867255	0.000159		0.859408	0.000152	0.862093	0.000148		0.858085	0.000276	0.859109	0.000289
			$R_{1}S_{3}$		0.865996	0.000152		0.859018	0.000151	0.861506	0.000147		0.859386	0.000221	0.860716	0.000221
			$R_{2}S_{1}$		0.864776	0.000186		0.858980	0.000174	0.861221	0.000168		0.859780	0.000214	0.861154	0.000217
			$R_{2}S_{2}$		0.866871	0.000182		0.858887	0.000173	0.861765	0.000165		0.858403	0.000281	0.859676	0.000287
			$R_{2}S_{3}$		0.866010	0.000167		0.858919	0.000165	0.861494	0.000162		0.859609	0.000236	0.860951	0.000240
			$R_{3}S_{1}$		0.866248	0.000146		0.860241	0.000144	0.862289	0.000134		0.858935	0.000262	0.860310	0.000261
			$R_{3}S_{2}$		0.866197	0.000168		0.858479	0.000165	0.861513	0.000154		0.858702	0.000269	0.859845	0.000275
			$R_{3}S_{3}$		0.866281	0.000158		0.859453	0.000157	0.861898	0.000155		0.858986	0.000253	0.860193	0.000257
	3	0.641039	$R_{1}S_{1}$		0.640959	0.000047		0.638244	0.000036	0.639038	0.000041		0.638535	0.000052	0.638723	0.000058
			$R_{1}S_{2}$		0.641791	0.000050		0.637976	0.000039	0.639163	0.000041		0.637944	0.000066	0.638198	0.000075
			$R_{1}S_{3}$		0.641321	0.000049		0.638020	0.000040	0.638895	0.000042		0.637812	0.000068	0.637992	0.000076
			$R_{2}S_{1}$		0.641044	0.000056		0.638206	0.000048	0.639052	0.000047		0.637898	0.000064	0.638300	0.000069
			$R_{2}S_{2}$		0.641344	0.000056		0.637496	0.000046	0.638652	0.000048		0.638055	0.000071	0.638288	0.000079
			$R_{2}S_{3}$		0.641637	0.000048		0.638195	0.000040	0.639189	0.000041		0.637932	0.000070	0.638069	0.000078
			$R_{3}S_{1}$		0.641066	0.000047		0.638348	0.000039	0.639217	0.000039		0.637996	0.000061	0.638369	0.000062
			$R_{3}S_{2}$		0.641587	0.000052		0.637820	0.000042	0.638937	0.000045		0.637820	0.000069	0.638724	0.000076
			$R_{3}S_{3}$		0.641357	0.000045		0.638012	0.000038	0.638981	0.000039		0.637741	0.000069	0.637925	0.000079

On comparing various results tabulated in Table 2, Table 3, Table 4, Table 5, we come up with following important observations:

• The reliability $R_{2,6}$ of a 2-out-of-6 system is higher than the reliability of $R_{3,6}$ of a 3-out-of-6 system in both the unknown as well as known η cases. A very obvious observation that a system which can work with lesser number of components has higher reliability.
• For unknown η, the point estimates obtained for $R_{2,6}$ and $R_{3,6}$ for the two sets of parameters $\theta_{1}$ and $\theta_{2}$ show that the MSEs and associated bias values for Bayesian estimates under informative priors are smaller than the classical ML estimates (see, Table 2). Similar results are also obtained for the two sets of parameters $\theta_{3}$ and $\theta_{4}$ in the known η case (see, Table 3).
• Further, comparing the results of Lindley and MCMC estimates leads to better performance of the Lindley estimates in terms MSEs and their bias values in all cases (see, Table 4, Table 5).
• In all the cases, the credible intervals perform better than the ACIs in terms of interval length and coverage probabilities i.e. Bayesian intervals have shorter average interval length and higher coverage probability (see, Table 4, Table 5).
• On comparing the Bayesian estimates under informative and non-informative prior assumptions, we see under informative prior the estimates perform better than those under non-informative prior in terms of their comparison criteria (see, Table 2, Table 3, Table 4, Table 5).
• It can also be observe that under non-informative prior assumptions the Bayesian point estimates are quite competitive but still perform better than the ML estimates. Similar result hold for the interval estimates. The informative credible intervals perform better than the non-informative credible intervals with smaller AILs and higher CPs (see, Table 2, Table 3, Table 4, Table 5).
• For the case when η is unknown, under the informative prior assumption, the CPs of credible intervals are quite close to the nominal value where as the CPs of the ACIs are little lower (see, Table 5).
• For the case when η is known, CPs of ACIs improve and is close to the nominal level. But in this case, for non-informative prior assumption, CPs of credible intervals is little below the nominal level (see, Table 5).
• A comparison among all the combinations of censoring schemes, we see the that the combination $\left(R_{1},S_{1}\right)$ performs better than the others and $\left(R_{3},S_{1}\right)$ is observed the next competing combination of censoring scheme in almost all of the cases. The point estimates under $\left(R_{1},S_{1}\right)$ show smaller MSEs and biases and the interval estimates have smaller interval length with CPs close to the nominal level.

**Table 3:** Classical and Bayesian point estimates and associated MSEs of $R_{s,k}$ when η is known.

$\theta_{i}$	s	$R_{s,k}$	CS		MLE			Informative prior					Non-informative prior			
					${\overset{ˆ}{R}}_{s,k}$	MSE		Lindley		MCMC			Lindley		MCMC	
								${\overset{ˆ}{R}}_{s,k}$	MSE	${\overset{ˆ}{R}}_{s,k}$	MSE		${\overset{ˆ}{R}}_{s,k}$	MSE	${\overset{ˆ}{R}}_{s,k}$	MSE
$\theta_{3}$	2	0.783320	$R_{1}S_{1}$		0.784040	0.001103		0.783434	0.000595	0.776663	0.000930		0.784202	0.000702	0.775295	0.001088
			$R_{1}S_{2}$		0.783078	0.001253		0.781862	0.000607	0.773316	0.001122		0.783807	0.000842	0.773852	0.001378
			$R_{1}S_{3}$		0.784434	0.001171		0.783209	0.000578	0.777160	0.000972		0.783041	0.000784	0.773248	0.001255
			$R_{2}S_{1}$		0.785938	0.001059		0.785030	0.000537	0.776043	0.000945		0.784144	0.000737	0.771797	0.001232
			$R_{2}S_{2}$		0.785035	0.001221		0.783519	0.000584	0.773590	0.001129		0.784068	0.000861	0.770810	0.001479
			$R_{2}S_{3}$		0.783610	0.001091		0.782771	0.000529	0.773107	0.001045		0.784318	0.000772	0.772516	0.001281
			$R_{3}S_{1}$		0.785844	0.001037		0.784948	0.000531	0.776607	0.000897		0.783886	0.000816	0.773433	0.001293
			$R_{3}S_{2}$		0.785423	0.001104		0.783638	0.000530	0.775434	0.000931		0.786265	0.000836	0.774509	0.001364
			$R_{3}S_{3}$		0.783478	0.001084		0.782558	0.000530	0.774505	0.000988		0.784895	0.000820	0.773748	0.001333
	3	0.599772	$R_{1}S_{1}$		0.601469	0.000184		0.602188	0.000094	0.598590	0.000150		0.601722	0.000160	0.596682	0.000224
			$R_{1}S_{2}$		0.601915	0.000219		0.602175	0.000109	0.598051	0.000160		0.601989	0.000159	0.596662	0.000242
			$R_{1}S_{3}$		0.601222	0.000203		0.601858	0.000203	0.598190	0.000166		0.602350	0.000169	0.597172	0.000241
			$R_{2}S_{1}$		0.600826	0.000209		0.601882	0.000115	0.597066	0.000166		0.601570	0.000155	0.595760	0.000230
			$R_{2}S_{2}$		0.601272	0.000239		0.6018051	0.000115	0.596099	0.000210		0.601687	0.000167	0.594402	0.000281
			$R_{2}S_{3}$		0.601516	0.000229		0.602185	0.000114	0.597503	0.000186		0.602565	0.000186	0.596532	0.000265
			$R_{3}S_{1}$		0.600934	0.000219		0.601860	0.000113	0.597474	0.000182		0.602474	0.000160	0.596783	0.000225
			$R_{3}S_{2}$		0.601459	0.000218		0.601914	0.000106	0.596960	0.000189		0.601490	0.000160	0.594838	0.000257
			$R_{3}S_{3}$		0.600991	0.000210		0.601688	0.000103	0.597301	0.000171		0.601660	0.000166	0.595979	0.000241
$\theta_{4}$	2	0.827286	$R_{1}S_{1}$		0.827660	0.000540		0.822413	0.000280	0.819835	0.000486		0.821778	0.000500	0.816322	0.000775
			$R_{1}S_{2}$		0.827630	0.000631		0.821494	0.000317	0.817952	0.000597		0.822434	0.000593	0.812987	0.001011
			$R_{1}S_{3}$		0.827086	0.000586		0.821397	0.000301	0.818499	0.000557		0.820482	0.000602	0.814452	0.000967
			$R_{2}S_{1}$		0.825904	0.000591		0.821399	0.000309	0.816689	0.000589		0.821585	0.000558	0.813604	0.000897
			$R_{2}S_{2}$		0.827383	0.000618		0.821525	0.000318	0.814934	0.000665		0.820354	0.000655	0.808614	0.001258
			$R_{2}S_{3}$		0.827900	0.000619		0.822038	0.000313	0.817387	0.000650		0.820434	0.000604	0.811400	0.001125
			$R_{3}S_{1}$		0.826145	0.000642		0.821498	0.000364	0.818534	0.000537		0.822784	0.000494	0.815332	0.000831
			$R_{3}S_{2}$		0.826115	0.000620		0.820370	0.000343	0.816210	0.000619		0.820675	0.000609	0.810282	0.001126
			$R_{3}S_{3}$		0.826524	0.000607		0.821205	0.000320	0.816929	0.000633		0.821271	0.000532	0.813583	0.000983
	3	0.620382	$R_{1}S_{1}$		0.619905	0.000141		0.618752	0.000062	0.616973	0.000112		0.619269	0.000113	0.615528	0.000177
			$R_{1}S_{2}$		0.621106	0.000133		0.619144	0.000055	0.616694	0.000122		0.618781	0.000111	0.613977	0.000206
			$R_{1}S_{3}$		0.621073	0.000144		0.619277	0.000061	0.617032	0.000119		0.619591	0.000115	0.615641	0.000184
			$R_{2}S_{1}$		0.620551	0.000143		0.619287	0.000064	0.616317	0.000128		0.619381	0.000120	0.614723	0.000196
			$R_{2}S_{2}$		0.620551	0.000146		0.618892	0.000061	0.618892	0.000146		0.618805	0.000130	0.612118	0.000271
			$R_{2}S_{3}$		0.620257	0.000147		0.618808	0.000064	0.615874	0.000135		0.619359	0.000118	0.614108	0.000216
			$R_{3}S_{1}$		0.619885	0.000152		0.618668	0.000078	0.616406	0.000123		0.619100	0.000110	0.614933	0.000186
			$R_{3}S_{2}$		0.620091	0.000149		0.618488	0.000065	0.614959	0.000146		0.619683	0.000122	0.614165	0.000229
			$R_{3}S_{3}$		0.619932	0.000139		0.618607	0.000060	0.615659	0.000130		0.619002	0.000119	0.614413	0.000211

**Table 4:** Interval estimates of $R_{s,k}$ when η is unknown.

θ	s	CS		ACI			Informative Bayes			Non-informative Bayes	
				AIL	CP		AIL	CP		AIL	CP
$\theta_{1}$	2	$R_{1}S_{1}$		0.141698	0.945		0.115798	0.953		0.123577	0.939
		$R_{1}S_{2}$		0.158781	0.951		0.122221	0.962		0.131537	0.925
		$R_{1}S_{3}$		0.150188	0.938		0.118355	0.947		0.126168	0.928
		$R_{2}S_{1}$		0.145513	0.922		0.116520	0.933		0.124411	0.917
		$R_{2}S_{2}$		0.159968	0.928		0.122604	0.934		0.131369	0.916
		$R_{2}S_{3}$		0.151388	0.928		0.119080	0.929		0.128301	0.928
		$R_{3}S_{1}$		0.144148	0.926		0.115571	0.932		0.125067	0.935
		$R_{3}S_{2}$		0.159026	0.928		0.121703	0.937		0.160093	0.929
		$R_{3}S_{3}$		0.150297	0.926		0.117995	0.932		0.127842	0.933
	3	$R_{1}S_{1}$		0.062865	0.949		0.050239	0.946		0.053554	0.927
		$R_{1}S_{2}$		0.069687	0.942		0.052999	0.952		0.056841	0.922
		$R_{1}S_{3}$		0.066681	0.949		0.051371	0.950		0.055165	0.936
		$R_{2}S_{1}$		0.064871	0.944		0.050607	0.949		0.054115	0.927
		$R_{2}S_{2}$		0.071421	0.954		0.053378	0.948		0.057352	0.927
		$R_{2}S_{3}$		0.067444	0.953		0.051790	0.946		0.055281	0.937
		$R_{3}S_{1}$		0.063625	0.953		0.050369	0.948		0.053828	0.926
		$R_{3}S_{2}$		0.070963	0.953		0.052960	0.956		0.056643	0.933
		$R_{3}S_{3}$		0.568150	0.954		0.051418	0.943		0.055070	0.929
$\theta_{2}$	2	$R_{1}S_{1}$		0.050253	0.917		0.045366	0.944		0.053233	0.916
		$R_{1}S_{2}$		0.057313	0.933		0.047735	0.948		0.059745	0.921
		$R_{1}S_{3}$		0.052844	0.922		0.047263	0.942		0.055077	0.932
		$R_{2}S_{1}$		0.051097	0.928		0.046478	0.925		0.052792	0.912
		$R_{2}S_{2}$		0.057398	0.925		0.048312	0.943		0.058896	0.917
		$R_{2}S_{3}$		0.054244	0.925		0.047696	0.947		0.054855	0.933
		$R_{3}S_{1}$		0.050792	0.931		0.044946	0.951		0.053321	0.903
		$R_{3}S_{2}$		0.055922	0.930		0.048690	0.946		0.058493	0.918
		$R_{3}S_{3}$		0.053349	0.917		0.046535	0.937		0.055949	0.921
	3	$R_{1}S_{1}$		0.029090	0.923		0.024874	0.947		0.028463	0.919
		$R_{1}S_{2}$		0.032462	0.938		0.026153	0.957		0.030965	0.916
		$R_{1}S_{3}$		0.030425	0.934		0.025735	0.955		0.030146	0.900
		$R_{2}S_{1}$		0.029882	0.916		0.024941	0.934		0.029127	0.913
		$R_{2}S_{2}$		0.032346	0.924		0.026787	0.949		0.312591	0.895
		$R_{2}S_{3}$		0.031018	0.933		0.025617	0.958		0.030278	0.900
		$R_{3}S_{1}$		0.029104	0.928		0.024796	0.959		0.028773	0.922
		$R_{3}S_{2}$		0.032540	0.936		0.026530	0.947		0.031325	0.918
		$R_{3}S_{3}$		0.030534	0.937		0.025775	0.972		0.030239	0.917

**Table 5:** Interval estimates of $R_{s,k}$ when η is known.

θ	s	CS		ACI			Informative Bayes			Non-informative Bayes	
				AIL	CP		AIL	CP		AIL	CP
$\theta_{3}$	2	$R_{1}S_{1}$		0.136630	0.934		0.115824	0.946		0.125090	0.942
		$R_{1}S_{2}$		0.143902	0.917		0.122469	0.927		0.132531	0.912
		$R_{1}S_{3}$		0.142835	0.927		0.118987	0.942		0.130097	0.924
		$R_{2}S_{1}$		0.141067	0.953		0.117595	0.939		0.127744	0.923
		$R_{2}S_{2}$		0.146606	0.934		0.123676	0.923		0.134804	0.909
		$R_{2}S_{3}$		0.143249	0.938		0.122279	0.944		0.131616	0.934
		$R_{3}S_{1}$		0.138760	0.946		0.116377	0.939		0.125852	0.911
		$R_{3}S_{2}$		0.146190	0.947		0.122640	0.946		0.132186	0.921
		$R_{3}S_{3}$		0.141906	0.934		0.120736	0.945		0.130572	0.909
	3	$R_{1}S_{1}$		0.061015	0.959		0.050002	0.951		0.053540	0.917
		$R_{1}S_{2}$		0.064518	0.955		0.052425	0.955		0.056776	0.918
		$R_{1}S_{3}$		0.063165	0.956		0.051465	0.945		0.055106	0.919
		$R_{2}S_{1}$		0.061368	0.957		0.050863	0.941		0.054258	0.912
		$R_{2}S_{2}$		0.065220	0.945		0.053076	0.932		0.057390	0.902
		$R_{2}S_{3}$		0.063992	0.955		0.051893	0.936		0.055664	0.908
		$R_{3}S_{1}$		0.061403	0.940		0.050068	0.916		0.053580	0.920
		$R_{3}S_{2}$		0.064331	0.946		0.052594	0.937		0.057024	0.906
		$R_{3}S_{3}$		0.063287	0.948		0.051762	0.949		0.055797	0.910
$\theta_{4}$	2	$R_{1}S_{1}$		0.101853	0.941		0.087271	0.951		0.100877	0.927
		$R_{1}S_{2}$		0.106407	0.927		0.092174	0.941		0.109567	0.908
		$R_{1}S_{3}$		0.104422	0.934		0.090493	0.945		0.105870	0.916
		$R_{2}S_{1}$		0.101266	0.932		0.090261	0.941		0.103434	0.917
		$R_{2}S_{2}$		0.106756	0.930		0.095261	0.947		0.113834	0.900
		$R_{2}S_{3}$		0.107216	0.930		0.091897	0.938		0.108558	0.887
		$R_{3}S_{1}$		0.101174	0.915		0.088304	0.954		0.101119	0.925
		$R_{3}S_{2}$		0.105408	0.920		0.093881	0.938		0.111792	0.914
		$R_{3}S_{3}$		0.104330	0.933		0.091689	0.930		0.106820	0.901
	3	$R_{1}S_{1}$		0.050043	0.928		0.041798	0.958		0.047550	0.918
		$R_{1}S_{2}$		0.052319	0.941		0.043748	0.945		0.051215	0.917
		$R_{1}S_{3}$		0.052556	0.942		0.042844	0.947		0.049220	0.918
		$R_{2}S_{1}$		0.050803	0.939		0.042513	0.935		0.048346	0.905
		$R_{2}S_{2}$		0.052726	0.934		0.044625	0.930		0.052124	0.871
		$R_{2}S_{3}$		0.052457	0.944		0.043801	0.942		0.050445	0.919
		$R_{3}S_{1}$		0.050240	0.924		0.042064	0.949		0.048113	0.915
		$R_{3}S_{2}$		0.052393	0.927		0.044560	0.941		0.051131	0.905
		$R_{3}S_{3}$		0.051351	0.932		0.043729	0.941		0.049971	0.908

## 6 Testing problem

In the section above, we have discussed about inferences of MSS reliability where stress and strength variables follow logistic distribution with common scale parameter η. Our interest here is to check equivalence of the scale parameter η with two different scale parameters for stress and strength variables $\eta_{1}$ and $\eta_{2}$ for illustration. Thus, we propose a hypothesis testing problem as discussed below:

$$H_{0}:\eta_{1}=\eta_{2}=\eta \;\text{versus}\;H_{1}:\eta_{1}\neq \eta_{2}.$$

For discussed MSS reliability problem, suppose $l_{1}\left(\overset{ˆ}{\lambda },\overset{ˆ}{\alpha },\overset{ˆ}{\eta }\right)$ denotes the log-likelihood with common scale parameter η. In the similar fashion, $l_{2}\left(\overset{ˆ}{\lambda },\overset{ˆ}{\alpha },\overset{ˆ}{\eta_{1}},\overset{ˆ}{\eta_{2}}\right)$ denotes the log-likelihood function with unequal scale parameters $\eta_{1}$ and $\eta_{2}$ respectively. Then, for sufficiently large value of n, the likelihood ratio statistic ($S_{LRT}$) is represented as

$$S_{LRT}=-2\left(l_{1}\left(\overset{ˆ}{\lambda },\overset{ˆ}{\alpha },\overset{ˆ}{\eta }\right)-l_{2}\left(\overset{ˆ}{\lambda },\overset{ˆ}{\alpha },\overset{ˆ}{\eta_{1}},\overset{ˆ}{\eta_{2}}\right)\right)\to \chi_{1}^{2}.$$

The likelihood ratio test can now be constructed using the asymptotic distribution of $S_{LRT}$ and reject $H_{0}$ if $S_{LRT}>c^{*}$ where $c^{*}$ is such that $P\left(\chi_{1}^{2}>c^{*}\right)$ = size of the test (ϵ).

## 7 Real data analysis

For illustration purposes, we study a numerical example in this section. The data set of our interest is 36 failure times of 500 MW generators recorded over a 6 year period by Dhillon [64]. The data set is given below:

**Table:** 

0.058	0.070	0.090	0.105	0.113	0.121	0.153	0.159	0.224	0.421	0.570	0.596
0.618	0.834	1.019	1.104	1.497	2.027	2.234	2.372	2.433	2.505	2.690	2.877
2.879	3.166	3.455	3.551	4.378	4.872	5.085	5.272	5.341	8.952	9.188	11.399

Since our study is based on MSS model, so we make the data in form of stress–strength by randomly splitting the data. A system containing 6 independent components is considered and working under some stress and the constructed data are tabulated as X and Y below. This set up provides an opportunity to study the failure pattern of a MSS system where simultaneous working of multiple generators working under some stress condition. This study can give a robust planning to prevent complete power failure scenarios. For example, in some govt programs with huge gathering may lead to some stamped or some other cascading effects due to complete failure of generator power system and similar application may be seen in big residential communities etc.

$$X=\left[\begin{array}{ccccc} 0.121 & 0.224 & 2.433 & 1.019 & 2.690\\ 0.105 & 4.872 & 1.497 & 3.166 & 2.879\\ 0.159 & 0.090 & 0.058 & 5.341 & 0.153\\ 4.378 & 2.877 & 3.551 & 3.455 & 0.596\\ 2.372 & 0.834 & 0.618 & 11.399 & 2.027\\ 1.104 & 0.113 & 2.234 & 8.952 & 5.272 \end{array}\right],\;\;Y=\left[\begin{array}{c} 0.570\\ 5.085\\ 9.188\\ 2.505\\ 0.421\\ 0.070 \end{array}\right]$$

**Fig. 4:**
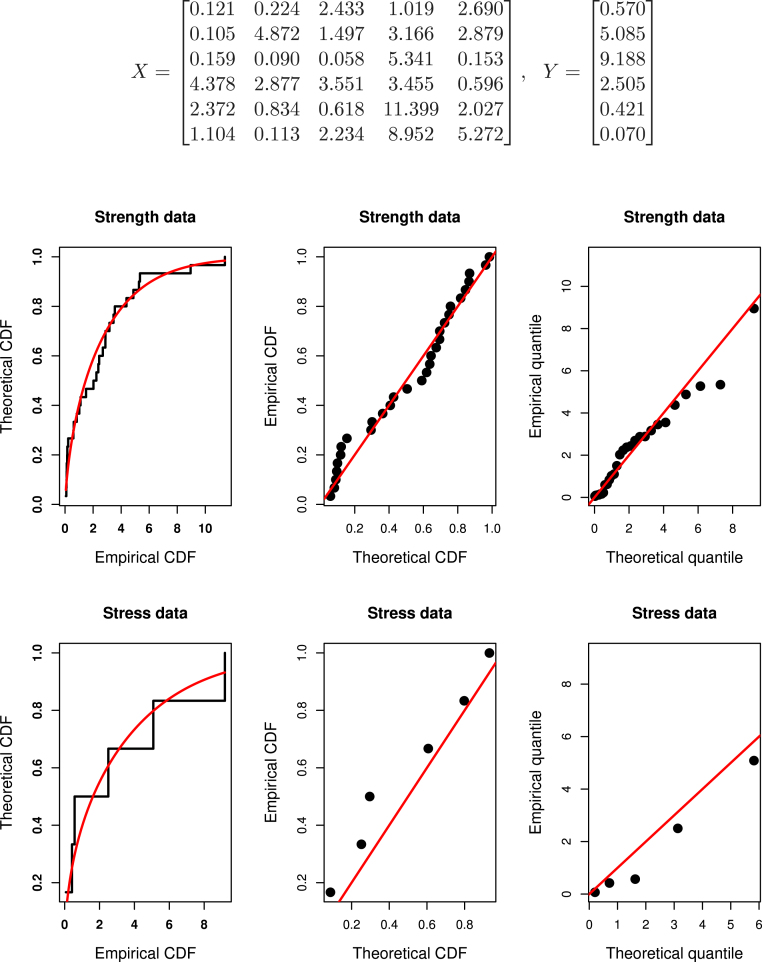
Theoretical–empirical CDF, P–P and Q–Q plot for goodness fit for real data.

Before we find various estimates under PCS set-up, it is an important task to check the adequacy of fitting of complete data to the considered statistical distributions. Therefore, the associated Kolmogorov–Smirnov (K–S) distances and P values are obtained for X and Y. Obtained K–S values are 0.1236 and 0.2034 and the P-values are 0.7031 and 0.9242 for X and Y, respectively. The K–S distances and P-values values indicate the two data fit the considered logistic-exponential distribution reasonably well. Furthermore, we created a theoretical and empirical CDF plot along with P–P and Q–Q plot in Fig. 4, which clearly demonstrates that the logistic-exponential distribution adequately fits the data set. As discussed in Section 6, we obtain the likelihood ratio test statistics $S_{LRT}$ to see if the null hypothesis $H_{0}:\eta_{1}=\eta_{2}=\eta$ gets accepted and the obtained value of $S_{LRT}$ suggests that the $H_{0}:\eta_{1}=\eta_{2}=\eta$ gets accepted. In turn, we may take the scale parameters $\eta_{1}$ and $\eta_{2}$ equal. Here, we consider estimation under two PCS data sets with schemes (1) CS1: R=(1,0,0,0),S=(1,0,0,0,0) and (2) CS2: R=(0,0,0,1),S=(0,0,0,2). The data under these two censoring schemes are obtained as

**Table 6:** Point and interval estimates of $R_{s,k}$ under censored real data.

(s,k)	CS	${\overset{ˆ}{R}}_{s,k}^{ML}$	${\overset{ˆ}{R}}_{s,k}^{Lin}$	${\overset{ˆ}{R}}_{s,k}^{MCMC}$		$ACI_{s,k}$	$CRI_{s,k}$
(1,4)	CS1	0.769996	0.786596	0.752450		(0.647920, 0.892072)	(0.636401, 0.874160)
	CS2	0.822524	0.824809	0.794020		(0.681492, 0.963556)	(0.655952, 0.904797)
(2,4)	CS1	0.586780	0.599505	0.597538		(0.534492, 0.639069)	(0.534072, 0.601474)
	CS2	0.610797	0.625820	0.615213		(0.540345, 0.681249)	(0.560284 0.661007)

$$X_{CS1}=\left[\begin{array}{cccc} 0.121 & 2.433 & 1.019 & 2.690\\ 0.159 & 0.058 & 5.341 & 0.153\\ 4.378 & 3.551 & 3.455 & 0.596\\ 2.372 & 0.618 & 11.399 & 2.027\\ 1.104 & 2.234 & 8.952 & 5.272 \end{array}\right],\;\;Y_{CS1}=\left[\begin{array}{c} 0.570\\ 9.188\\ 2.505\\ 0.421\\ 0.070 \end{array}\right]$$

and

$$X_{CS2}=\left[\begin{array}{cccc} 0.121 & 0.224 & 2.433 & 1.019\\ 0.105 & 4.872 & 1.497 & 3.166\\ 0.159 & 0.090 & 0.058 & 5.341\\ 4.378 & 2.877 & 3.551 & 3.455 \end{array}\right],\;\;Y_{CS2}=\left[\begin{array}{c} 0.570\\ 5.085\\ 9.188\\ 2.505 \end{array}\right]$$

**Fig. 5:**
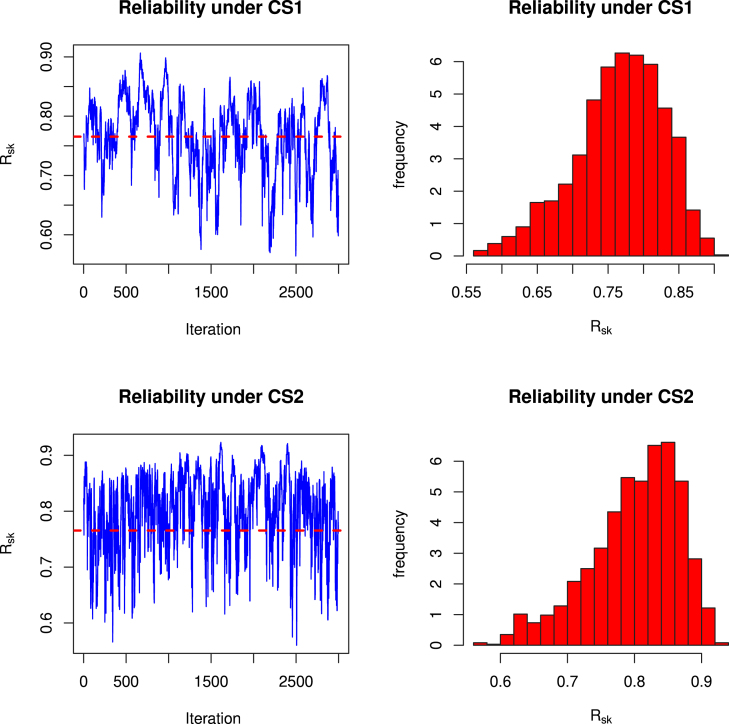
MCMC trace and histogram plot for $R_{s,k}$ when (s,k)=(1,4).

**Fig. 6:**
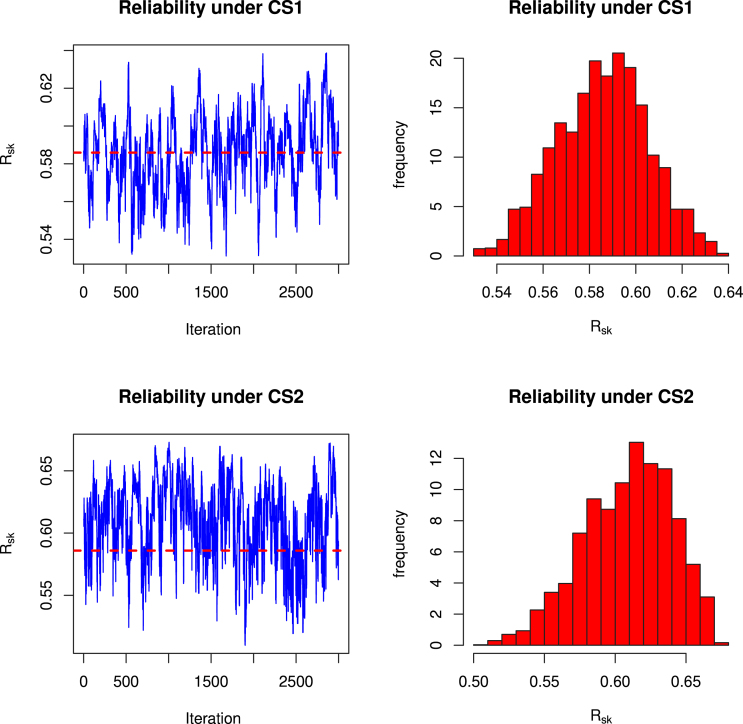
MCMC trace and histogram plot for $R_{s,k}$ when (s,k)=(2,4).

For both the schemes, we obtain the estimates of ${\overset{ˆ}{R}}_{1,4}$ and ${\overset{ˆ}{R}}_{2,4}$ under classical as well as Bayesian set up. The Bayesian point and interval estimates are obtained under consideration of non informative priors by setting very small values for the hyperparameters. Widely used trace plots and histograms are good visualization technique to visualize the convergence of MCMC algorithm. Considering this, for assessing the convergence of the MCMC algorithm for the considered multicomponent data sets two sets of (s,k) values, we present trace plots of 3000 MCMC samples for $R_{s,k}$ in Fig. 5, Fig. 6. From the plots, we observed satisfactory convergence for all parameters under considered multicomponent data sets. Therefore, the trace plot and histogram suggest that good convergence of MCMC algorithms. The Bayesian estimates are obtained under the non-informative prior assumption. All the point and interval estimates under classical as well as Bayesian methods are tabulated in Table 6.

We see that results obtained under two censored real data sets coincide with the performance of results of simulation studies. Point and interval estimates under Bayesian set are quite similar to those of classical estimation methods. The length of credible intervals is smaller compare to those of ACIs.

## 8 Conclusions

In this article, we have considered estimation of MSS reliability under progressively censored data. The stress and strength components of the multicomponent system are considered to be following logistic exponential distributions. Under assumption that the common parameter for the stress and strength components is unknown, various classical and Bayesian estimates are obtained. The likelihood estimation is used to obtain the point estimates in the classical estimation and the asymptotic confidence interval is constructed. Bayesian point estimates are obtained using Lindley and MCMC methods. The credible intervals are also constructed from MCMC samples. The Bayesian point and interval estimates are obtained under informative and non-informative prior assumptions. The various estimates discussed are also obtained for the case with known common shape parameter. Extensive simulations are conducted and associated observations are made. It is observed that the Bayesian point intervals perform better than the classical point estimates in terms of MSEs and biases for both the cases considered. The credible intervals perform better than the ACIs in terms of AIL and CPs, and we also observed that for informative credible intervals the CPs are close to nominal level but for ACIs, CPs are a little lower. Furthermore, to illustrate the methods discussed, a real data set is also analyzed and similar observations are made.

## CRediT authorship contribution statement

**Amulya Kumar Mahto:** Data curation, Conceptualization. **Kumar Abhishek:** Conceptualization. **Yogesh Mani Tripathi:** Conceptualization. **Oluwafemi Samson Balogun:** Data curation. **Yusra A. Tashkandy:** Data curation, Conceptualization. **Mahmoud E. Bakr:** Data curation.

## Research involving human participants and/or animals

No human and/or animal participation in conducting the experiment is involved.

## Funding

The study was funded by Ongoing Research Funding program, (ORF-2025-1004), King Saud University, Riyadh, Saudi Arabia

## Declaration of competing interest

The authors declare that they have no known competing financial interests or personal relationships that could have appeared to influence the work reported in this paper.

## References

[1] Birnbaum Z.W. (1956). Proceedings of the Third Berkeley Symposium on Mathematical Statistics and Probability, : Contributions To the Theory of Statistics.

[2] Mukherjee S.P., Sharan L.K. (1985). Estimation of failure probability from a bivariate normal stress–strength distribution. Microelectron. Reliab..

[3] Hanagal D.D. (1997). Note on estimation of reliability under bivariate Pareto stress–strength model. Statist. Papers.

[4] Rao G.S., Rosaiah K., Babu M.S. (2016). Estimation of stress–strength reliability from exponentiated Fréchet distribution. Int. J. Adv. Manuf. Technol..

[5] Qayoom D., Rather A.A., Alqasem O.A., Ahmad Z., Nagy M., Yousuf A.M., Mansi A.H., Hussam E., Gemeay A.M. (2025). Development of a novel extension of Rayleigh distribution with application to COVID-19 data. Sci. Rep..

[6] Fulment A.K., Josephat P.K., Rao G.S. (2017). Estimation of reliability in multicomponent stress-strength based on dagum distribution. Stoch. Qual. Control..

[7] Ahmad A., Rather A.A., Alqasem O.A., Bakr M.E., Mekiso G.T., Balogun O.S., Hussam E., Gemeay A.M. (2025). Introducing novel arc cosine-class of distribution with theory and data evaluation related to coronavirus. Sci. Rep..

[8] Wang B.X., Geng Y., Zhou J.X. (2018). Inference for the generalized exponential stress–strength model. Appl. Math. Model..

[9] Hussam E., ALMetwally E.M. (2025). Statistical inference on progressive-stress accelerated life testing for the Perk distribution under adaptive type-II hybrid censoring scheme. J. Stat. Theory Appl..

[10] Kohansal A., Nadarajah S. (2019). Stress–strength parameter estimation based on Type-II hybrid progressive censored samples for a Kumaraswamy distribution. IEEE Trans. Reliab..

[11] Hussam E., Sapkota L.P., Gemeay A.M. (2024). Tangent exponential-g family of distributions with applications in medical and engineering. Alex. Eng. J..

[12] Rezaei A., Yousefzadeh F., Jomhoori S. (2019). Estimation of stress–strength reliability for the multivariate SGPII distribution. Comm. Statist. Theory Methods.

[13] Alosey A.R.E., Gemeay A.M. (2025). A novel version of geometric distribution: Method and application. Comput. J. Math. Stat. Sci..

[14] Saini S., Chaturvedi A., Garg R. (2021). Estimation of stress–strength reliability for generalized Maxwell failure distribution under progressive first failure censoring. J. Stat. Comput. Simul..

[15] Ragab I.E., Elgarhy M. (2025). Type II half logistic ailamujia distribution with numerical illustrations to medical data. Comput. J. Math. Stat. Sci..

[16] BuHamra S.S., Al-Kandari N.M., Hussam E., Almetwally E.M., Gemeay A.M. (2024). A case study for Kuwait mortality during the consequent waves of COVID-19. Heliyon.

[17] Agiwal V. (2023). Bayesian estimation of stress strength reliability from inverse Chen distribution with application on failure time data. Ann. Data Sci..

[18] Alghamdi F.M., Ahsan-ul Haq M., Hussain M.N.S., Hussam E., Almetwally E.M., Aljohani H.M., Mustafa M.S., Alshawarbeh E., Yusuf M. (2024). Discrete Poisson Quasi-XLindley distribution with mathematical properties, regression model, and data analysis. J. Radiat. Res. Appl. Sci..

[19] Kumari R., Lodhi C., Tripathi Y.M., Sinha R.K. (2023). Estimation of stress–strength reliability for inverse exponentiated distributions with application. Int. J. Qual. Reliab. Manag..

[20] El-Alosey A.R., Alotaibi M.S., Gemeay A.M. (2024). A new two-parameter mixture family of generalized distributions: Statistical properties and application. Heliyon.

[21] Asadi S., Panahi H., Anwar S., Lone S.A. (2023). Reliability estimation of burr type III distribution under improved adaptive progressive censoring with application to surface coating. Eksploat. I Niezawodność.

[22] Tashkandy Y.A., Almetwally E.M., Ragab R., Gemeay A.M., Abd El-Raouf M.M., Khosa S.K., Hussam E., Bakr M.E. (2023). Statistical inferences for the extended inverse Weibull distribution under progressive type-II censored sample with applications. Alex. Eng. J..

[23] Sindhu T.N., Anwar S., Hassan M.K., Lone S.A., Abushal T.A., Shafiq A. (2023). An analysis of the new reliability model based on bathtub-shaped failure rate distribution with application to failure data. Mathematics.

[24] Hassan A.S., Morgan Y.S. (2024). Stress–strength reliability inference for exponentiated half-logistic distribution containing outliers. Qual. Quant..

[25] Moheb S., Hassan A.S., Diab L.S. (2024). Classical and Bayesian inferences of stress–strength reliability model based on record data. Commun. Stat. Appl. Methods.

[26] Milošević B., Stanojević J. (2024). On the estimation of fuzzy stress–strength reliability parameter. J. Comput. Appl. Math..

[27] Shafiq A., Sindhu T.N., Lone S.A., Abushal T.A., Hassan M.K. (2024). An updated software reliability model using the shanker model and failure data. Qual. Reliab. Eng. Int..

[28] Lone S.A., Panahi H., Anwar S., Shahab S. (2024). Inference of reliability model with burr type XII distribution under two sample balanced progressive censored samples. Phys. Scr..

[29] Bhattacharyya G.K., Johnson R.A. (1974). Estimation of reliability in a multicomponent stress–strength model. J. Amer. Statist. Assoc..

[30] Rao G.S., Aslam M., Kundu D. (2015). Burr-XII distribution parametric estimation and estimation of reliability of multicomponent stress–strength. Comm. Statist. Theory Methods.

[31] Kizilaslan F., Nadar M. (2015). Classical and Bayesian estimation of reliability in multicomponent stress–strength model based on Weibull distribution. Rev. Colomb. de Estad..

[32] Nadar M., Kızılaslan F. (2015). Estimation of reliability in a multicomponent stress–strength model based on a Marshall–Olkin bivariate Weibull distribution. IEEE Trans. Reliab..

[33] Dey S., Mazucheli J., Anis M.Z. (2017). Estimation of reliability of multicomponent stress–strength for a Kumaraswamy distribution. Comm. Statist. Theory Methods.

[34] Kızılaslan F., Nadar M. (2018). Estimation of reliability in a multicomponent stress–strength model based on a bivariate Kumaraswamy distribution. Statist. Papers.

[35] Kızılaslan F. (2018). Classical and Bayesian estimation of reliability in a multicomponent stress–strength model based on a general class of inverse exponentiated distributions. Statist. Papers.

[36] Dey S., Moala F.A. (2019). Estimation of reliability of multicomponent stress–strength of a bathtub shape or increasing failure rate function. Int. J. Qual. Reliab. Manag..

[37] Singh D.P., Jha M.K., Tripathi Y., Wang L. (2022). Reliability estimation in a multicomponent stress–strength model for unit Burr III distribution under progressive censoring. Qual. Technol. Quant. Manag..

[38] Saini S., Tomer S., Garg R. (2022). On the reliability estimation of multicomponent stress–strength model for Burr XII distribution using progressively first-failure censored samples. J. Stat. Comput. Simul..

[39] Wang L., Wu K., Tripathi Y.M., Lodhi C. (2022). Reliability analysis of multicomponent stress–strength reliability from a bathtub-shaped distribution. J. Appl. Stat..

[40] Akgul F.G. (2023). Estimation of multicomponent stress–strength reliability based on unit Burr XII distribution: an application to dam occupancy rate of Istanbul, Turkey. J. Stat. Comput. Simul..

[41] Zhu T. (2024). Reliability inference for multicomponent stress–strength model under generalized progressive hybrid censoring. J. Comput. Appl. Math..

[42] Singh K., Kumar Mahto A., Mani Tripathi Y., Wang L. (2024). Estimation in a multicomponent stress–strength model for progressive censored lognormal distribution. Proc. Inst. Mech. Eng. Part O: J. Risk Reliab..

[43] Singh K., Mahto A.K., Tripathi Y., Wang L. (2024). Inference for reliability in a multicomponent stress–strength model for a unit inverse Weibull distribution under type-II censoring. Qual. Technol. Quant. Manag..

[44] Kumari T., Pathak A. (2024). Efficient estimation of the reliability functions in a multicomponent stress–strength set-up for a generalized family of distributions under progressive type II censoring. Qual. Reliab. Eng. Int..

[45] Kohansal A., Pérez-González C.J., Fernández A.J. (2024). Inference on the stress–strength reliability of multi-component systems based on progressive first failure censored samples. Proc. Inst. Mech. Eng. Part O: J. Risk Reliab..

[46] Kohansal A. (2019). On estimation of reliability in a multicomponent stress–strength model for a Kumaraswamy distribution based on progressively censored sample. Statist. Papers.

[47] Kayal T., Tripathi Y.M., Dey S., Wu S.J. (2019). On estimating the reliability in a multicomponent stress–strength model based on Chen distribution. Comm. Statist. Theory Methods.

[48] Maurya R.K., Tripathi Y.M. (2020). Reliability estimation in a multicomponent stress–strength model for Burr XII distribution under progressive censoring. Braz. J. Probab. Stat..

[49] Jha M.K., Dey S., Alotaibi R.M., Tripathi Y.M. (2020). Reliability estimation of a multicomponent stress–strength model for unit Gompertz distribution under progressive Type II censoring. Qual. Reliab. Eng. Int..

[50] Mahto A.K., Dey S., Mani Tripathi Y. (2020). Statistical inference on progressive-stress accelerated life testing for the logistic exponential distribution under progressive type-II censoring. Qual. Reliab. Eng. Int..

[51] A.K. Mahto, Y.M. Tripathi, Estimation of reliability in a multicomponent stress–strength model for inverted exponentiated Rayleigh distribution under progressive censoring, OPSEARCH 57 (4).

[52] Kohansal A., Shoaee S., Nadarajah S. (2022). Multi-component stress–strength model for Weibull distribution in progressively censored samples. Stat. Risk Model..

[53] Lan Y., Leemis L.M. (2008). The logistic–exponential survival distribution. Naval Res. Logist..

[54] Myers M.H., Hankey B.F., Mantel N. (1973). A logistic-exponential model for use with response-time data involving regressor variables. Biometrics.

[55] Chatterjee S., Singh J.B. (2014). A NHPP based software reliability model and optimal release policy with logistic–exponential test coverage under imperfect debugging. Int. J. Syst. Assur. Eng. Manag..

[56] van Staden P.J., King R.A. (2016). Kurtosis of the logistic-exponential survival distribution. Comm. Statist. Theory Methods.

[57] Dutta S., Kayal S. (2022). Estimation of parameters of the logistic exponential distribution under progressive type-Ihybrid censored sample. Qual. Technol. Quant. Manag..

[58] Olapade A.K. (2022). The type I generalized half logistic distribution. J. Iran. Stat. Soc..

[59] Dey S., Saha M., Anis M.Z., Maiti S.S., Kumar S. (2023). Estimation and confidence intervals of C Np (u, v) for logistic-exponential distribution with application. Int. J. Syst. Assur. Eng. Manag..

[60] Kohansal A. (2017). On estimation of reliability in a multicomponent stress–strength model for a Kumaraswamy distribution based on progressively censored sample. Statist. Papers.

[61] Lindley D.V. (1980). Approximate bayesian methods. Trab. Estadística Y Investig. Oper..

[62] Ahmed E.A. (2014). Bayesian estimation based on progressive Type-II censoring from two-parameter bathtub-shaped lifetime model: an Markov chain Monte Carlo approach. J. Appl. Stat..

[63] Chen M.H., Shao Q.M. (1999). Monte Carlo estimation of Bayesian credible and HPD intervals. J. Comput. Graph. Statist..

[64] Dhillon B.S. (1981). Life distributions. IEEE Trans. Reliab..

